# Computational Identification and Systematic Classification of Novel Cytochrome P450 Genes in *Salvia miltiorrhiza*

**DOI:** 10.1371/journal.pone.0115149

**Published:** 2014-12-10

**Authors:** Haimei Chen, Bin Wu, David R. Nelson, Kai Wu, Chang Liu

**Affiliations:** 1 Center for Bioinformatics, Institute of Medicinal Plant Development, Chinese Academy of Medical Sciences and Peking Union Medical College, Beijing, China; 2 Department of Microbiology, Immunology and Biochemistry, University of Tennessee Health Science Center, Memphis, Tennessee, United States of America; National Institute of Plant Genome Research, India

## Abstract

*Salvia miltiorrhiza* is one of the most economically important medicinal plants. Cytochrome P450 (CYP450) genes have been implicated in the biosynthesis of its active components. However, only a dozen full-length CYP450 genes have been described, and there is no systematic classification of CYP450 genes in *S. miltiorrhiza*. We obtained 77,549 unigenes from three tissue types of *S. miltiorrhiza* using RNA-Seq technology. Combining our data with previously identified CYP450 sequences and scanning with the CYP450 model from Pfam resulted in the identification of 116 full-length and 135 partial-length CYP450 genes. The 116 genes were classified into 9 clans and 38 families using standard criteria. The RNA-Seq results showed that 35 CYP450 genes were co-expressed with CYP76AH1, a marker gene for tanshinone biosynthesis, using r≥0.9 as a cutoff. The expression profiles for 16 of 19 randomly selected CYP450 obtained from RNA-Seq were validated by qRT-PCR. Comparing against the KEGG database, 10 CYP450 genes were found to be associated with diterpenoid biosynthesis. Considering all the evidence, 3 CYP450 genes were identified to be potentially involved in terpenoid biosynthesis. Moreover, we found that 15 CYP450 genes were possibly regulated by antisense transcripts (r≥0.9 or r≤–0.9). Lastly, a web resource (SMCYP450, http://www.herbalgenomics.org/samicyp450) was set up, which allows users to browse, search, retrieve and compare CYP450 genes and can serve as a centralized resource.

## Introduction

*Salvia miltiorrhiza* Bunge (Chinese name: Danshen) is a perennial plant belonging to the Lamiaceae family. The root of *S. miltiorrhiza* has been used as a traditional Chinese medicine in the treatment of dysmenorrhea, amenorrhea, cardiovascular diseases, blood circulation disturbances, inflammation, and angina pectoris [1]. The active components of *S. miltiorrhiza* consist of water-soluble and lipid-soluble compounds. The lipid-soluble tanshinones, such as tanshinone I, tanshinone IIA, tanshinone IIB, and cryptotanshinone, are abietane-type norditerpenoid naphthoquinones, which are considered as the main lipophilic bioactive components of *S. miltiorrhiza* [2]. The annual sales of medicinal products containing active components of *S. miltiorrhiza* exceed US$ 1 billion. Due to the medical and economic importance of tanshinones, increasing the production of these compounds through marker-assisted breeding and metabolic engineering has become an active area of research. Accordingly, the identification of all genes involved in the biosynthesis of tanshinones and the elucidation of the corresponding pathways are critical for achieving these goals.

The putative biosynthetic pathway of tanshinones can be roughly divided into three stages [3]. In the first stage, the end products are isopentenyl diphosphate (IPP) or its isomer dimethylallyl diphosphate (DMAPP), which are synthesized mainly in plastids via the 2-C-methyl-D-erythritol 4-phosphate (MEP) pathway and/or seldom in the cytosol or peroxisomes via the mevalonic acid (MEV) pathway [3], [4]. In the second stage, IPP is converted to geranyl diphosphate (GPP), farnesyl diphosphate (FPP) and geranylgeranyl diphosphate (GGPP), which are the precursors of monoterpenoids, sesquiterpenoids and diterpenoids, respectively [5]. In the third stage, GGPP is used to produce the bicyclic labdane-type diterpenoids via stepwise ionization and cycloisomerization [6]. To our knowledge, copalyl diphosphate synthase (CPS), kaurene synthase-like synthase (KSL), Cytochrome P450-dependent monooxygenases (CYP450s), various types of transferases (e.g., acyl-, aryl-, methyl-, or glycosyl-transferases) and oxidoreductases have been hypothesized to be involved in this stage [7]. So far, only three steps in the third stage have been elucidated. First, GGPP is converted into copalyl diphosphate by SmCPS1 [8]. Then, SmKSL1 converts copalyl diphosphate into miltiradiene [8]. Finally, CYP76AH1 and CYP76AH4 hydroxylate the facilely oxidized aromatic intermediate abietatriene [9].

In recent years, the function identification of CYP450 genes has become a hot area of research for understanding the downstream pathways of tanshinones. So far, five studies focusing on the transcriptome-wide identification of CYP450 genes for tanshinone biosynthesis in *S. miltiorrhiza* have been reported. One earlier study performed transcriptomic analyses using 454 pyrosequencing data from roots, leading to the discovery of 70 CYP450 unigenes [10]. Subsequently, approximately 300 isotigs showing similarity to CYP450 genes have been identified from hairy roots using the RNA-Seq technology, of which six were studied in detail and the CYP76AH1 gene was functionally characterized [11]. Additionally, the transcriptome profiling of *S. miltiorrhiza* root and leaf tissues has been conducted using the 454 GS-FLX pyrosequencing platform [12], yielding the discovery of 63 CYP450 unigenes with significant differential expression between leaf and root tissues. In addition, 15 CYP450 genes that were likely to contain complete open reading frames were confirmed using the 5′ and 3′ RACE method. Phylogenetic analysis of CYP450 proteins from *S. miltiorrhiza* and those from other plants revealed that 9 of them belong to the CYP71 clan, 3 to the CYP85 clan, and the remaining 3 to the CYP72 clan. In a more recent study [13], 125 expressed CYP450 genes belonging to 8 clans and 31 families were studied using RNA-Seq data. Among them, the expression profiles of 8 CYP450 genes were co-regulated with some already identified enzymatic genes (e.g., SmCPS). Most recently, the transcriptional profiling of *S. miltiorrhiza* leaves at 12 h after MeJA elicitation and mock-treatment using the RNA-Seq technology identified 122 CYP450 genes. Among them, 3 CYP450 genes were found to potentially to be involved in terpenoid biosynthesis [14]. These studies demonstrated the wide interest in the CYP450 genes of *S. miltiorrhiza*.

The CYP450 genes are a large family of genes that play multiple roles in many critical cellular processes [15]. The CYP450 genes are notorious for their difficulty in classification and naming, as the family has a large number of genes with diverse levels of sequence similarities [16]–[19]. Without a standard nomenclature, it is difficult to compare CYP450 genes belonging to the same species but identified in different studies and those derived from different species. Despite the five transcriptome-wide efforts attempting to identify CYP450 genes from *S. miltiorrhiza*, only a dozen full-length CYP450 genes could be retrieved from international EST and protein sequence databases. Furthermore, there is no well-curated set of CYP450 gene sequences and a systematic nomenclature available for the research community focusing on *S. miltiorrhiza.* Actually, the seemingly redundant studies described above reflect the fact that the information regarding the sequences and classification of CYP450 genes identified from earlier studies are fragmented and difficult to access. A well-curated set of CYP450 genes from *S. miltiorrhiza* and a standard nomenclature are direly needed for the identification and characterization of CYP450 genes that might be involved in the biosynthesis of active components in *S. miltiorrhiza.*

Our goal is to establish a centralized resource of CYP450 genes in *S. miltiorrhiza*, which is critical for the functional characterization of these genes. As the whole genome sequence of *S. miltiorrhiza* is not available and many of the sequences reported in previous transcriptome-wide studies were not accessible, we conducted another large-scale RNA-Seq experiment using samples from three tissues. We first compared the assembled transcript sequences and the CYP450 gene sequences that have been described previously. Those with significant similarities were merged, and the merged sequences were then manually curated to determine if they encoded the full-length proteins. Then, the sequences were classified according to the well-accepted CYP450 gene naming conventions. Next, the CYP450 gene set was mined to identify genes that might be involved in terpenoid biosynthesis, the main type of active compound in *S. miltiorrhiza*. Lastly, an on-line resource was set up, which makes the nomenclature and curated sequences for CYP450 genes easily accessible to the research community. The sequences and on-line resource produced from this study will be beneficial for future studies on CYP450 genes in *S. miltiorrhiza*.

## Materials and Methods

### Plant Materials and RNA extraction

Two-year-old *S. miltiorrhiza* Bunge *cv* 99-3 plants were grown in the field of the Beijing Medicinal Plant Garden of the Institute of Medicinal Plant Development, Chinese Academy of Medical Sciences and Peking Union Medical College (Beijing, China). Fresh leaves, roots and flowers were collected from three individual *S. miltiorrhiza* plants at the full-bloom stage on May 12, 2013 and immediately stored at −80°C until use. Total RNA was extracted from each tissue sample using the RNAprep pure plant kit (Tiangen, China) according to the manufacturer’s protocol, which can eliminate genomic DNA contamination during the course of extraction.

### RNA-Seq library construction and sequencing

Three RNA samples from the same tissue type were pooled and used for strand-specific RNA-Seq library construction. After mRNA was isolated from total RNA with Sera-Mag Magnetic Oligo(dT) particles, the mRNA was chemically fragmented. The sequence library construction was carried out according to the ScriptSeq mRNA-Seq Library Preparation Kit (Illumina-compatible). The resulting cDNA was cleaved into small fragments (300 bp to 400 bp) to construct sequencing libraries, followed by emulsion PCR and sequencing according to the manufacturer’s protocols.

#### Sequence assembly and annotation

The RNA-Seq reads were assembled using Trinity software [20] with the parameter ‘-SS_lib_type FR’ option, which allows the specification of the library type to be FR, indicating that the first read of the fragment pair is sequenced in the sense strand, and the second read is sequenced in the antisense strand. This provided a strand-specific and reference independent assembly. To annotate the assembled transcripts, we compared the *ab initio* assembly with known sequences in the SWISS-PROT, NR and NT databases using BLAST with an E-value cutoff of 1e-5. In parallel, the transcripts were categorized by searching them against the Kyoto Encyclopedia of Genes and Genomes (KEGG) database [21] to obtain the KO numbers and KEGG reference metabolic pathways. Only hits having the same orientation as the query transcripts were kept for downstream analyses.

### Computational identification of full-length CYP450 genes in *S. miltiorrhiza*

The CYP450 genes of *S. miltiorrhiza* were identified as described below. First, we searched the Pfam database (http://pfam.sanger.ac.uk/) with the keyword ‘cytochrome P450’, and a total of 39,592 sequences were identified and subsequently downloaded to construct a local CYP450 reference database. The 137,856 transcript sequences of *S. miltiorrhiza* were searched against the local reference database using the BLASTX algorithm, with an e-value cutoff of 1e-5. For each unigene cluster having matches, the transcript sequence having the lowest e-value was selected as the representative of the unigene. Second, *S. miltiorrhiza* sequences reported as similar to CYP450 in previous studies were downloaded from international DNA and protein sequence databases to construct a local database. The unigene sequences obtained above were compared with these sequences using the sequence identity >97% and the length of the aligned region >300 nucleotides as the cutoffs. The selected transcripts were subjected to open reading frame (ORF) identification using the GETORF program (EMBOSS, Version 6.4). The ORFs were used to search the Pfam database at http://pfam.sanger.ac.uk/, with an e-value cutoff of 1e-5. Third, the ORFs matching the HMM model (PF00067) were then used to search the CYPED database (http://www.cyped.uni-stuttgart.de/cgi-bin/blast/doblastcyped5.pl), with an e-value cutoff of 1e-5. Up to ten best-hit homologous sequences found in CYPED were selected and subjected to multiple sequence alignment. Fourth, the alignment was manually inspected and edited accordingly. Lastly, the full-length CYP450 proteins were identified based on the two following criteria: (1) the corresponding protein starts with amino acid “M” and stops before a position corresponding to a stop codon, and (2) the amino acid sequence before the predicted starting amino acid “M” is not conserved compared to the corresponding regions of the homologous sequences (http://www.herbalgenomics.org/samicyp450/msa/index_msa.html).

### Classification and characterization of full-length *S. miltiorrhiza* CYP450 genes

All the full-length CYP450 proteins were named according to the standard CYP450 nomenclature [22] using sequences from a well-annotated CYP450 database [23] as reference sequences. In particular, 40%, 55% and 97% sequence identities were used as cutoffs for family, subfamily and allelic variants, respectively. If a sequence did not match any reference CYP450 sequences with identity >40%, it was discarded as contamination from non-plant sources.

Then, the name of the CYP450 proteins was assigned by Prof. David Nelson. The sequences were divided into A-type, which includes only the CYP71 clan, and non-A-type, which includes all other clans, based on CYP family membership. All full-length CYP450 proteins were subjected to motif analyses using the Multiple Expectation Maximization for Motif Elicitation (MEME) program [24] and Motif Alignment and Search Tool (MAST) [25] for the motifs CYP450 cysteine heme-iron ligand signature, PERF motif, K-helix region and I helix region [26]. The sequences 5 bp up- and downstream of the conserved sequences were extracted and used to create sequence logos using WEBLOGO (WEBLOGO.berkeley.edu).

The theoretical iso-electric point and molecular weight for each identified CYP450 proteins were predicted using the “Compute pI/MW” tool on the ExPASy server [27]. The localizations of deduced proteins were predicted on the TargetP 1.1 server with specificity >0.95 [28].

### Phylogenetic analysis of predicted CYP450 proteins

The sequences of 271 *S. lycopersicum* CYP450 proteins and the 116 full-length *S. miltiorrhiza* CYP450 proteins were retrieved. Multiple sequence alignment was performed using the MUSCLE module included in the MEGA6 package [29]. The phylogenetic tree was constructed using the Neighbor-Joining (NJ) method with “Poisson correction” and “pairwise deletion of gaps” in MEGA6. The significance level for the phylogenetic tree was assessed by bootstrap testing with 1000 replications. Only branches supported by bootstrap values >50% are shown.

### Expression analysis of CYP450 genes using RNA-Seq data

The expression levels of CYP450 unigenes were calculated using the Trinity program [30] and represented as numbers of fragments per kilobase per million fragments mapped (FPKM). The unigenes having a FPKM ≤1 were considered as not expressed. The FPKM was added to 1, log transformed and centered with the mean of the expression levels of three different tissues and subjected to hierarchical analysis using JMP (Version 10, SAS, North Carolina).

### Validation of RNA-Seq data by qRT-PCR

For each tissue type, the RNA samples extracted from three individuals were used as biological replicates for qRT-PCR analyses. Each biological replicate had three technical replicates. For each sample, reverse transcription was performed on 2 µg total RNA by 200 U M-MLV Transcriptase (Takara) in a 20 µl volume. The reaction was carried out at 70°C for 10 minutes, 42°C for 60 minutes and 70°C for 15 minutes. The resulting cDNA was diluted to 800 µl with sterile water. qPCR was carried out in triplicate reactions using an ABI 7500 Fast instrument (Applied Biosystems). Gene-specific primers were designed using PrimerQuest (http://www.idtdna.com/Primerquest/Home/Index). The primers used in this study are listed in S1 Table. The Actin gene (Accession no.: ADK11998) was chosen as an endogenous control. PCR was carried out in a 20 µl volume containing 2 µl diluted cDNA, 250 nM forward primer, 250 nM reverse primer, and 1×SYBR Premix Ex Taq II (TaKaRa) using the following conditions: 95°C for 3 min and 40 cycles of 95°C for 15 sec, 60°C for 15 sec and 72°C for 15 sec. Melting curve analyses were performed to verify the specificity by ABI 7500 Fast instrument Manage software. The relative expression levels were calculated using the 2–^ΔΔCt^ method [31].

### Co-expression of genes among tissues

The Pearson correlation coefficients between the expression profiles of CYP450 genes, SmCPS1, and SmKSL1 and that of the reference gene SmCYP76AH1 were calculated using JMP software (Version 10, SAS, North Carolina).

### Identification of antisense RNAs corresponding to full length CYP450 genes

To obtain the candidate antisense RNA (asRNA) related to the 116 full length CYP450 genes, the coding sequences (CDS) of the 116 full length CYP450 genes were searched against the 137,856 transcript sequences of *S. miltiorrhiza* using the BLASTN algorithm, with an E-value cutoff of 1e-5. The candidate asRNAs were identified based on the four following criteria: (1) query sequences found on the opposite strand of the CYP450 genes; (2) alignment length ≥100 bp; (3) an E-value cutoff of 1e-50; and (4) identity ≥99%.

## Results

### Illumina paired-end sequencing and *de* *novo* assembly

**Figure 1:**
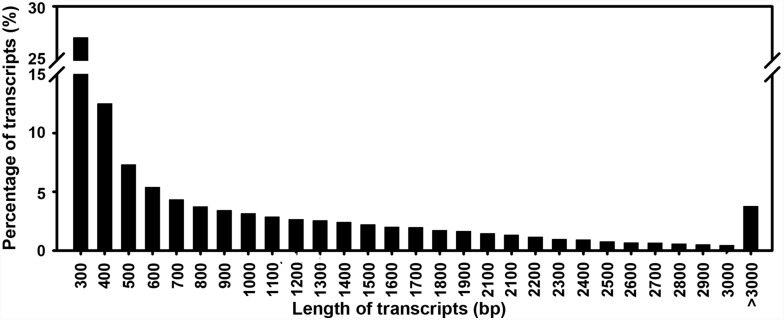
Sequence length distribution of the assembled transcripts. The X-axis shows the range of lengths of the transcript sequences. The Y-axis shows the percentage of transcripts.

A total of 25.3, 53.8 and 26.7 million 100-bp paired end reads were generated from the flower, leaf and root samples, respectively. Then, the unigenes were assembled from the pooled reads of the three tissues using Trinity [30]. The resulting assembly contained 77,549 unigenes. As some unigenes contained multiple alternatively spliced transcripts, there were a total of 137,856 unique transcripts. The length of these transcripts ranged from 201 to 12,362 bp, with an average length of 933 bp (Fig. 1). The RNA-Seq data can be accessed from NCBI with the accession numbers SRR1043988, SRR1045051 and SRR1020591.

#### Annotation of *de* *novo* transcriptome

**Figure 2:**
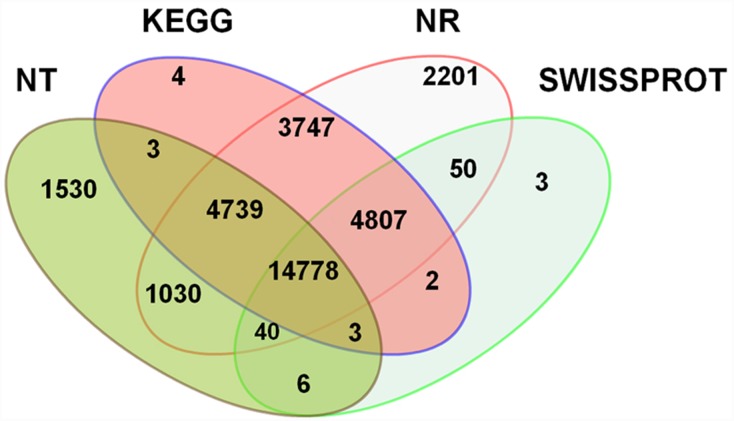
Venn diagram indicating annotated genes by the KEGG, NR, NT and SwissProt databases. The number of genes annotated is listed in each diagram component.

The longest transcript in each unigene was selected as the representative transcript. These transcripts were searched against the Nt, Nr, SWISS-PROT and the KEGG databases for annotation. The results indicated that 28,083 (36.2%), 31,392 (40.5%), 22,129 (28.5%) and 19,689 (25.4%) unigenes showed significant similarity to known sequences in the KEGG, Nr, Nt, and SWISS-PROT databases, respectively (Fig. 2). Taken together, 32,943 (42.5%) unigenes were annotated by the four databases.

### Identification and classification of CYP450 genes in *S. miltiorrhiza*

In total, we identified 116 full-length and 135 partial CYP450 genes in *S. miltiorrhiza* in this study. The total number of distinct full-length and partial-length CYP450 genes was 251, which is comparable to that for *A. thaliana* (246) but lower than that for *V. vinifera* (315). However, without the whole genome sequence of *S. miltiorrhiza*, the percentage of all *S. miltiorrhiza* CYP450 genes that this number, 251, represents cannot be determined. Manual examination of the multiple sequence alignment was used to determine whether a sequence encoded the full-length protein. All the alignments used in this exercise can be accessed from http://www.herbalgenomics.org/samicyp450. The aligned regions between 113 of the 116 full-length CYP450 proteins and the CYP450 HMM model (PF00067) covered more than 85% of the model length. The exceptions were SmCYP74A1, SmCYP74B21 and SmCYP90A39 (S2 Table). The SmCYP74A1 and SmCYP74B21 proteins seemed to contain only part of the entire CYP450 Pfam domain. However, the examination of its alignment with its homologous sequences suggests that they are full-length proteins. Furthermore, in comparison with the CYP450 HMM model from Pfam, the domain in SmCYP90A39 seems to have been disrupted, likely by an insertion. Among the 135 partial CYP450 proteins, the length of the aligned regions of 131 CYP450 protein sequences was less than 80% of the length for the CYP450 HMM model. The alignment regions of four sequences were longer than 80% of that of the HMM model. However, they were considered partial, as they did not have stop codons (S3 Table). Then, the 135 partial CYP450s were assembled with previously published ESTs in Genbank. However, we did not identify any new full-length CYP450 proteins. Additional experiments are needed to obtain the full-length of these partial proteins.

The classification of the 116 full-length CYP450 genes was conducted by comparing them with those in the Cytochrome P450 database [23] using the standard sequence similarity cutoffs, specifically 97% for allelic variant, 55% for subfamily and 40% for family. Based on these criteria, the 116 full-length CYP450s were classified to 38 families and 69 subfamilies (Table 1). Among them, eight CYP450s have been previously reported. The other 108 CYP450s were identified for the first time in *S. miltiorrhiza*. We then compared the distribution of CYP450 genes among *S. miltiorrhiza*, *A. thali*ana, *S. lycopersicum*, and V. *vinifera*. The 116 full-length CYP450 genes were categorized as either A-type (CYP71 clan) or non- A-type (all other clans) and are shown in Table 2 and Table 3, respectively. For the A-type, the largest family in *S. miltiorrhiza* is CYP71, containing 19 members. Members from CYP92 and CYP736 were found in *S. lycopersicum* and V. *vinifera* but not in *A. thali*ana. For the non-A-type, the largest families in *S. miltiorrhiza* were CYP94 and CYP72, and each contained 6 members. Members from CYP728 were found in three of the species but were not found in *A. thali*ana. In contrast, members from CYP749 were found in *S. miltiorrhiza* and *S. lycopersicum* but not in *A. thali*ana and V. *vinifera.* Lastly, members from CYP749, CYP728 and CYP727 were not found in *A. thali*ana and *S. lycopersicum,* even though a small number of them were found in *S. miltiorrhiza and* V. *vinifera*. The fact that these families can be missing from intact genomes indicates that they most likely do not perform essential functions.

**Table 1:** List of full-length CYP450s of *S. miltiorrhiza* identified in this study.

No.	GeneName	Type	CYPClan	CYPFamily	CYPSubfamily	AccessionNo^a^	Length	PI	Mol.wt (kDa)	Loc^b^
1	SmCYP98A75	A	71	CYP98	CYP98A	TBA	509	8.96	57.9	S
2	SmCYP73A120	A	71	CYP73	CYP73A	TBA	504	9.11	57.7	S
3	SmCYP93B25	A	71	CYP93	CYP93B	TBA	510	8.59	57.4	*
4	SmCYP98A76	A	71	CYP98	CYP98A	TBA	512	8.55	58.0	S
5	SmCYP98A77	A	71	CYP98	CYP98A	TBA	508	8.88	57.2	*
6	SmCYP51G1	non-A	51	CYP51	CYP51G	TBA	492	8.09	55.3	S
7	SmCYP701A40	A	71	CYP701	CYP701A	TBA	519	7.22	58.8	S
8	SmCYP704A98	non-A	86	CYP704	CYP704A	TBA	511	6.74	59.2	S
9	SmCYP704B37	non-A	86	CYP704	CYP704B	TBA	506	7.16	58.2	S
10	SmCYP704A99	non-A	86	CYP704	CYP704A	TBA	506	6.9	57.7	S
11	SmCYP706C35	A	71	CYP706	CYP706C	TBA	518	8.17	57.7	*
12	SmCYP706G11	A	71	CYP706	CYP706G	TBA	507	9.06	56.8	*
13	SmCYP707A99	non-A	85	CYP707	CYP707A	TBA	488	9.27	55.1	S
14	SmCYP707A100	non-A	85	CYP707	CYP707A	TBA	481	9.45	54.9	*
15	SmCYP707A101	non-A	85	CYP707	CYP707A	TBA	479	9.34	54.9	S
16	SmCYP707A102	non-A	85	CYP707	CYP707A	TBA	470	8.6	53.0	S
17	SmCYP711A44	non-A	711	CYP711	CYP711A	TBA	514	9.37	57.6	S
18	SmCYP714A25	non-A	72	CYP714	CYP714A	TBA	527	8.64	59.1	S
19	SmCYP714E21	non-A	72	CYP714	CYP714E	TBA	523	8.71	58.5	S
20	SmCYP716A89	non-A	85	CYP716	CYP716A	TBA	479	8.76	54.0	S
21	SmCYP716C12	non-A	85	CYP716	CYP716C	TBA	476	9	53.4	S
22	SmCYP728D17	non-A	85	CYP728	CYP728D	TBA	483	8.9	55.4	S
23	SmCYP716D25	non-A	85	CYP716	CYP716D	TBA	477	9.13	54.3	S
24	SmCYP71AU51	A	71	CYP71	CYP71AU	TBA	505	6.62	56.4	*
25	SmCYP71AU52	A	71	CYP71	CYP71AU	TBA	500	6.66	56.4	*
26	SmCYP71AH15	A	71	CYP71	CYP71AH	TBA	496	6.05	55.5	S
27	SmCYP71AP14	A	71	CYP71	CYP71AP	TBA	522	6.14	58.7	*
28	SmCYP71A57	A	71	CYP71	CYP71A	TBA	497	8.47	55.8	*
29	SmCYP71A58	A	71	CYP71	CYP71A	TBA	505	6.33	56.9	S
30	SmCYP71A59	A	71	CYP71	CYP71A	TBA	500	6.27	56.0	*
31	SmCYP71D410	A	71	CYP71	CYP71D	TBA	504	7.65	56.5	S
32	SmCYP71D411	A	71	CYP71	CYP71D	TBA	498	7.2	56.6	S
33	SmCYP71D374	A	71	CYP71	CYP71D	TBA	502	8.73	57.1	*
34	SmCYP71BE37	A	71	CYP71	CYP71BE	TBA	498	8.34	56.8	*
35	SmCYP71D412	A	71	CYP71	CYP71D	TBA	502	6.7	57.2	*
36	SmCYP71D413	A	71	CYP71	CYP71D	TBA	511	6.14	58.2	*
37	SmCYP720A1	non-A	85	CYP720	CYP720A	TBA	494	8.97	56.5	S
38	SmCYP72A326	non-A	72	CYP72	CYP72A	TBA	518	9.3	59.1	S
39	SmCYP72A327	non-A	72	CYP72	CYP72A	TBA	518	9.04	59.1	S
40	SmCYP72A328	non-A	72	CYP72	CYP72A	TBA	517	9.01	59.6	S
41	SmCYP72A329	non-A	72	CYP72	CYP72A	TBA	512	9.03	58.1	S
42	SmCYP72A330	non-A	72	CYP72	CYP72A	TBA	517	9.1	59.1	S
43	SmCYP72A331	non-A	72	CYP72	CYP72A	TBA	516	8.72	59.0	S
44	SmCYP734A33	non-A	72	CYP734	CYP734A	TBA	522	9.1	59.6	S
45	SmCYP749A37	non-A	72	CYP749	CYP749A	TBA	508	8.95	58.3	S
46	SmCYP749A38	non-A	72	CYP749	CYP749A	TBA	508	9.25	58.4	S
47	SmCYP721A38	non-A	72	CYP721	CYP721A	TBA	506	8.93	58.3	S
48	SmCYP749A39	non-A	72	CYP749	CYP749A	TBA	511	9.14	58.5	S
49	SmCYP749A40	non-A	72	CYP749	CYP749A	TBA	509	9	57.9	S
50	SmCYP727B10	non-A	727	CYP727	CYP727B	TBA	502	8.59	56.4	S
51	SmCYP714G13	non-A	72	CYP714	CYP714G	TBA	506	9.34	56.4	S
52	SmCYP714G14	non-A	72	CYP714	CYP714G	TBA	514	9.16	57.5	S
53	SmCYP736A121	A	71	CYP736	CYP736A	TBA	496	6.72	56.4	*
54	SmCYP736A122	A	71	CYP736	CYP736A	TBA	502	6.82	57.1	*
55	SmCYP736A123	A	71	CYP736	CYP736A	TBA	488	7.07	55.3	*
56	SmCYP74A1	non-A	74	CYP74	CYP74A	TBA	519	8.88	58.0	C
57	SmCYP74B21	non-A	74	CYP74	CYP74B	TBA	483	5.91	53.9	*
58	SmCYP75B79	A	71	CYP75	CYP75B	TBA	511	7.73	56.2	*
59	SmCYP75B80	A	71	CYP75	CYP75B	TBA	514	7.3	56.5	*
60	SmCYP92B28	A	71	CYP92	CYP92B	TBA	501	6.55	56.5	S
61	SmCYP75A57	A	71	CYP75	CYP75A	TBA	516	8.43	57.6	S
62	SmCYP92A73	A	71	CYP92	CYP92A	TBA	509	8.93	57.9	*
63	SmCYP92B29	A	71	CYP92	CYP92B	TBA	509	6.69	57.7	*
64	SmCYP76AH1	A	71	CYP76	CYP76AH	TBA	495	7.73	55.5	*
65	SmCYP76AK2	A	71	CYP76	CYP76AK	TBA	494	8.72	55.8	*
66	SmCYP76S7	A	71	CYP76	CYP76S	TBA	494	7.67	55.8	*
67	SmCYP76AK3	A	71	CYP76	CYP76AK	TBA	491	8.85	55.4	*
68	SmCYP76T27	A	71	CYP76	CYP76T	TBA	486	8.83	55.0	*
69	SmCYP76A35	A	71	CYP76	CYP76A	TBA	503	8.25	57.8	*
70	SmCYP76A36	A	71	CYP76	CYP76A	TBA	513	8.9	57.7	*
71	SmCYP77A27	A	71	CYP77	CYP77A	TBA	512	9.13	57.6	S
72	SmCYP77A28	A	71	CYP77	CYP77A	TBA	505	9.16	56.8	*
73	SmCYP78A113	A	71	CYP78	CYP78A	TBA	511	8.61	57.3	S
74	SmCYP78A114	A	71	CYP78	CYP78A	TBA	533	9.24	59.1	*
75	SmCYP78A115	A	71	CYP78	CYP78A	TBA	526	9.1	57.9	S
76	SmCYP79D40	A	71	CYP79	CYP79D	TBA	522	8.98	58.7	S
77	SmCYP81Q40	A	71	CYP81	CYP81Q	TBA	493	7.3	55.8	*
78	SmCYP81B61	A	71	CYP81	CYP81B	TBA	510	8.9	58.1	S
79	SmCYP81B62	A	71	CYP81	CYP81B	TBA	498	7.68	55.9	S
80	SmCYP81Q41	A	71	CYP81	CYP81Q	TBA	494	7.68	56.0	*
81	SmCYP81Q42	A	71	CYP81	CYP81Q	TBA	493	6.27	56.1	*
82	SmCYP81Q43	A	71	CYP81	CYP81Q	TBA	494	7.68	56.0	*
83	SmCYP81C16	A	71	CYP81	CYP81C	TBA	498	8.07	55.8	S
84	SmCYP82V2	A	71	CYP82	CYP82V	TBA	534	6.61	60.2	S
85	SmCYP82D70	A	71	CYP82	CYP82D	TBA	516	8.9	57.6	*
86	SmCYP82D71	A	71	CYP82	CYP82D	TBA	516	6.59	57.4	S
87	SmCYP82U4	A	71	CYP82	CYP82U	TBA	522	7.71	58.8	S
88	SmCYP71AT89	A	71	CYP71	CYP71AT	TBA	499	8.87	56.1	*
89	SmCYP71AT90	A	71	CYP71	CYP71AT	TBA	496	7.69	55.6	*
90	SmCYP71AT91	A	71	CYP71	CYP71AT	TBA	506	8.44	56.7	*
91	SmCYP71AT92	A	71	CYP71	CYP71AT	TBA	506	6.15	57.4	S
92	SmCYP71AT93	A	71	CYP71	CYP71AT	TBA	491	9.05	55.3	*
93	SmCYP84A60	A	71	CYP84	CYP84A	TBA	517	5.68	58.0	S
94	SmCYP84A61	A	71	CYP84	CYP84A	TBA	517	6.61	58.0	S
95	SmCYP85A1	non-A	85	CYP85	CYP85A	TBA	463	8.88	53.3	S
96	SmCYP86A91	non-A	86	CYP86	CYP86A	TBA	555	8.48	62.3	S
97	SmCYP86A92	non-A	86	CYP86	CYP86A	TBA	525	8.28	59.6	S
98	SmCYP96A84	non-A	86	CYP96	CYP96A	TBA	485	8.96	55.0	S
99	SmCYP96A85	non-A	86	CYP96	CYP96A	TBA	502	8.42	57.8	*
100	SmCYP88A52	non-A	85	CYP88	CYP88A	TBA	487	9.26	56.2	S
101	SmCYP89A115	A	71	CYP89	CYP89A	TBA	500	7.66	57.7	S
102	SmCYP90A39	non-A	85	CYP90	CYP90A	TBA	478	9.12	54.4	*
103	SmCYP90B26	non-A	85	CYP90	CYP90B	TBA	476	9.05	53.9	S
104	SmCYP90C19	non-A	85	CYP90	CYP90C	TBA	488	8.4	55.5	S
105	SmCYP94A48	non-A	86	CYP94	CYP94A	TBA	502	8.85	57.7	S
106	SmCYP94A49	non-A	86	CYP94	CYP94A	TBA	502	8.6	57.1	S
107	SmCYP94B50	non-A	86	CYP94	CYP94B	TBA	505	8.89	56.1	S
108	SmCYP94C54	non-A	86	CYP94	CYP94C	TBA	491	7.63	55.1	S
109	SmCYP94C55	non-A	86	CYP94	CYP94C	TBA	488	8.45	56.2	S
110	SmCYP94D47	non-A	86	CYP94	CYP94D	TBA	501	9.02	57.1	*
111	SmCYP97A41	non-A	97	CYP97	CYP97A	TBA	612	5.89	68.6	C
112	SmCYP97B34	non-A	97	CYP97	CYP97B	TBA	582	6.31	65.0	C
113	SmCYP97C28	non-A	97	CYP97	CYP97C	TBA	542	6.12	60.1	C
114	SmCYP98A78	A	71	CYP98	CYP98A	TBA	508	8.48	57.6	S
115	SmCYP76G16	A	71	CYP76	CYP76G	TBA	508	8.5	57.0	*
116	SmCYP71AU53	A	71	CYP71	CYP71AU	TBA	498	9.37	56.6	*

a TBA: To Be Added.

b Cellular location of the protein predicted using the TargetP program. ‘C’: chloroplast; ‘S’: secreted; ‘*’: unknown.

**Table 2:** Distribution of A-type full-length CYP450 genes among *A. thaliana* (At), *S. lycopersicum* (Sl), V. *vinifera* (Vv) and *S. miltiorrhiza* (Sm).

Family	At	Sl	Vv	Sm
CYP71	52	47	24	19
CYP73	1	3	3	1
CYP75	1	2	11	3
CYP76	8	22	24	8
CYP77	5	3	2	2
CYP78	6	6	7	3
CYP79	7	5	9	1
CYP80	0	5	6	0
CYP81	18	12	21	7
CYP82	5	9	34	4
CYP83	2	0	0	0
CYP84	2	1	3	2
CYP89	7	4	14	1
CYP92	0	8	6	3
CYP93	1	1	4	1
CYP98	3	5	1	4
CYP701	1	1	1	1
CYP703	1	1	1	0
CYP705	26	0	0	0
CYP706	7	11	9	2
CYP712	2	1	2	0
CYP736	0	11	8	3

**Table 3:** Distribution of non-A-type full-length CYP450 genes among *A. thaliana* (At), *S. lycopersicum* (Sl), V. *vinifera* (Vv) and *S. miltiorrhiza* (Sm).

Family	At	Sl	Vv	Sm
CYP51	1	1	2	1
CYP72	9	25	22	6
CYP709	3	0	1	0
CYP714	2	3	6	4
CYP715	1	1	1	0
CYP721	1	2	5	1
CYP734	1	3	2	1
CYP735	1	2	1	0
CYP749	0	2	0	4
CYP74	2	7	7	2
CYP85	2	2	2	1
CYP87	1	4	7	0
CYP88	2	4	2	1
CYP90	4	4	4	3
CYP702	6	0	0	0
CYP707	4	4	5	4
CYP708	4	0	0	0
CYP716	2	6	15	3
CYP718	1	1	0	0
CYP720	1	1	1	1
CYP722	1	2	1	0
CYP724	1	2	2	0
CYP728	0	1	6	1
CYP733	0	1	1	0
CYP86	11	4	6	2
CYP94	6	11	9	6
CYP96	13	7	5	2
CYP704	3	8	6	3
CYP97	3	3	3	3
CYP710	4	1	1	0
CYP711	1	1	1	1
CYP727	0	0	1	1

### Characterization of CYP450 proteins

The length, isoelectric point (pI), molecular weight and presence of signal peptide of the 116 CYP450 were characterized in detail (Table 1). The lengths of the 116 CYP450 proteins range from 463 to 612 amino acids, with an average length of 506 amino acids. The majority of CYP450 (67/116) protein sequences contained SP, a secondary pathway signal peptide. Only four CYP450 protein sequences (SmCYP74A1, SmCYP97B34, SmCYP97A41 and SmCYP97C28) contained chloroplast-targeting peptides, indicating that they most likely function in the chloroplast. In contrast, no CYP450 proteins were found to have mitochondrial targeting peptides. Previous studies have suggested that most CYP450s are anchored on the surface of the endoplasmic reticulum [32] and that only a few of them may target plastids or mitochondria [15]. In fact, so far, no CYP450s have been reported to be located in the mitochondria in plants. Our results are consistent with the current knowledge regarding the cellular locations of CYP450s.

### Phylogenetic analysis

**Figure 3:**
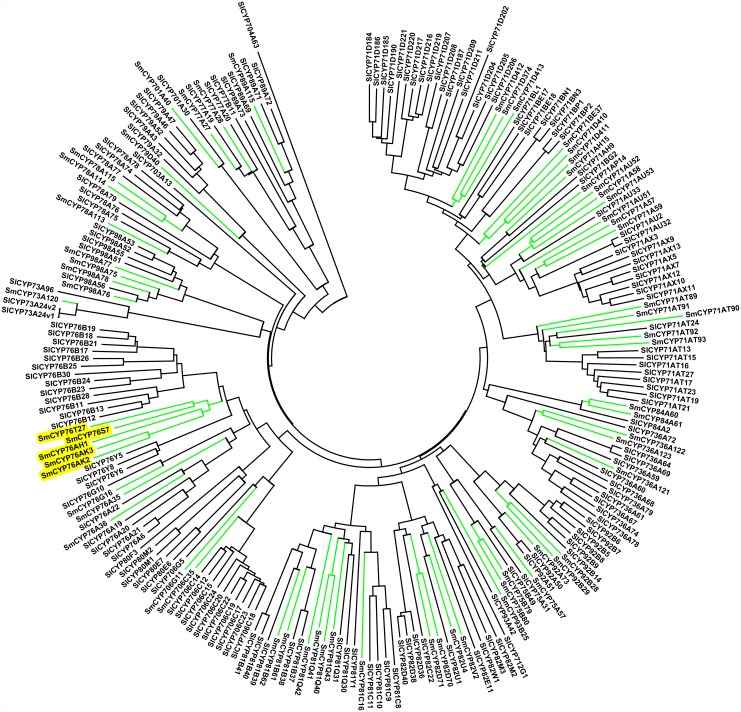
Phylogenetic analyses of A-type CYP450s from *S. lycopersicum* and *S. miltiorrhiza*. The Neighbor-Joining tree was constructed using the MEGA6 package. The spokes corresponding to Sm and Sl CYP450s are shown in green and black, respectively. The shaded genes are described in the text. Sm: *S. miltiorrhiza* and Sl: *S. lycopersicum*.

**Figure 4:**
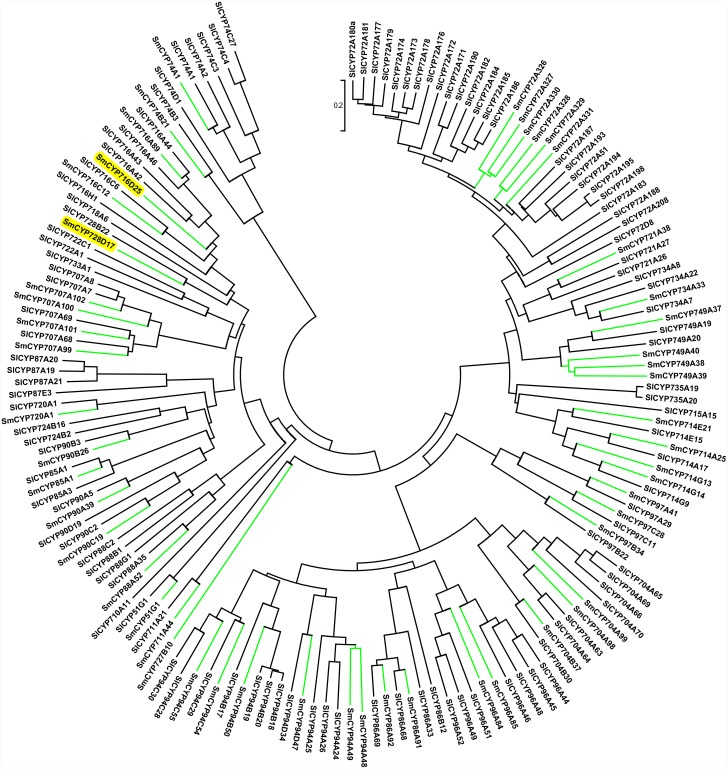
Phylogenetic analyses of non-A-type CYP450s from *S. lycopersicum* and *S. miltiorrhiza*. The Neighbor-Joining tree was constructed using the MEGA6 package. The spokes corresponding to Sm and Sl CYP450s are shown in green and black, respectively. The shaded genes are described in the text. Sm: *S. miltiorrhiza* and Sl: *S. lycopersicum*.

As described above, members from several families, such as CYP92A, CYP736, CYP749, CYP728 and CYP727, were missing in *A. thali*ana, suggesting that *A. thali*ana is not the best reference for *S. miltiorrhiza* in comparative studies of CYP450 genes. [29]. Instead, the *Solanum lycopersicum* CYPome was chosen for comparison because *S. lycopersicum* belongs to Solanales, the order closest to Lamiales, which includes the family lamiaceae and has well-annotated CYP450 genes. The sequences of 271 *S. lycopersicum* CYP450 proteins and the 116 full-length *S. miltiorrhiza* CYP450 proteins were used to construct Neighbor-Joining (NJ) trees for A-type (Fig. 3) and non- A-type (Fig. 4) CYP450s, respectively, using the MEGA6 program. The trees showed that among the 116 full-length CYP450 genes, 65 (56.0%) were A-type and distributed into 17 families. The remaining 51 (44.0%) full-length CYP450s were non-A-type, belonging to 21 families and 9 clans. Genes belonging to the CYP71 clan have been found to be involved in the biosynthesis of secondary metabolites or natural products [15], [33]. It has been reported that CYP76AH1 gene is involved in tanshinone biosynthesis [11]. The large number of subfamilies found in the CYP76 family indicates a diversity of functions, in which even genes within the same subfamily may have unique functions. The phylogenetic tree for non-A-type CYP450s is shown in Fig. 4. These include a much more divergent group of sequences covering the 8 remaining clans, which function in sterol, carotenoid, oxylipin, fatty acid and hormone metabolism [34] as well as secondary metabolite synthesis. It should be noted that most genes belonging to family CYP76 are clustered on different branches for *S. miltiorrhiza* and *S. lycopersicum* (Fig. 3, shaded area), suggesting that this family underwent independent expansion after the divergence of Lamiales and Solanales, possibly to meet unique functional needs.

### Conserved motifs in the 116 predicted CYP450 proteins

**Figure 5:**
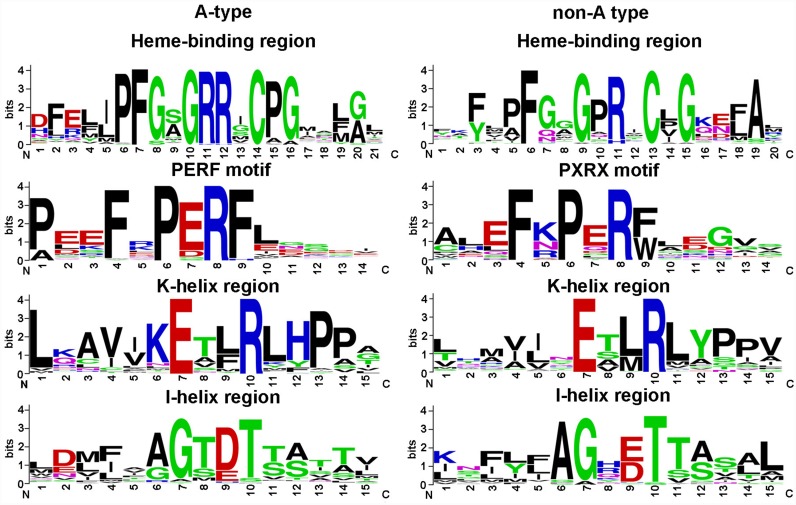
Weblogos of conserved motifs identified in the 116 CYP450s from *S. miltiorrhiza.* The names of the motifs are shown above each logo.

All CYP450 protein sequences have four distinct characteristics, including the cytochrome P450 cysteine heme-iron ligand signature motif, the PERF motif, the conserved EXXR motif located in the K-helix, and the I-helix, which contains a highly conserved threonine involved in oxygen activation [26], [35]. S. *miltiorrhiza* CYP450 proteins were subjected to MEME analysis to identify these motifs. The identified motifs were aligned using MAST and presented using WEBLOGO (Fig. 5). The consensus sequences of the heme-binding regions of A-type and non-A-type CYP450s are “PFGXGRRXCXG” and “XFXXGXRXCXG”, respectively. Similarly, the PERF motifs of A-type and non-A-type CYP450s are also different in their consensus sequences, “PERF” and “PXRX”, respectively [36]. In *S. miltiorrhiza*, the conserved motifs are likely to be “FXPERF” and “FXPXRX” for A-type and non-A-type sequences, respectively, having one additional highly conserved amino acid “F” at the −2 position of the previously described PERF motifs. However, the functional importance of this amino acid remains to be determined. The K-helix motifs of A-type and non-A-type CYP450s are similar to each other, with the sequence “EXXR”. In contrast, the I-helix motifs of A-type and non-A-type CYP450s are likely to be “AGXDT” and “AGX (E/D) T”. The consensus sequences of these motifs are similar to those that have previously been described.

### KEGG pathway analyses

**Figure 6:**
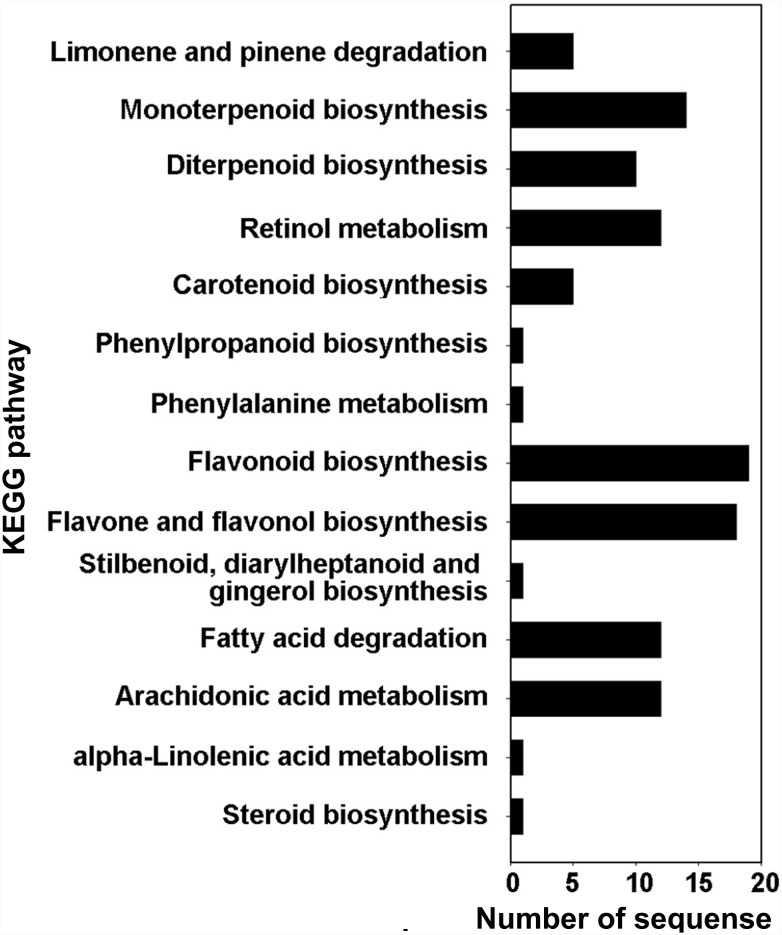
KEGG pathway analyses of predicted CYP450 genes in *S. miltiorrhiza.* The numbers of CYP450 genes involved in the corresponding metabolic processes are shown.

Pathway-based analysis was performed, as described in the methods section, to further understand the biological functions and interactions of the CYP450 genes. In total, 49 (44.2%) CYP450 proteins were assigned to 14 KEGG pathways, of which two were the monoterpenoid and diterpenoid biosynthesis pathways (Fig. 6). Particularly, 14 CYP450s were found to be involved in the monoterpenoid biosynthesis, including SmCYP71D410, SmCYP71D411, SmCYP71D374, SmCYP71D412, SmCYP71D413, SmCYP71AU52, SmCYP71A59, SmCYP71A57, SmCYP71A58, SmCYP71AU53, SmCYP81B62, SmCYP98A75, SmCYP98A76 and SmCYP98A77. Among them, 10 belonged to the CYP71 family, 3 belonged to the CYP98 family and 1 belonged to the CYP81 family. In contrast, 10 CYP450s were mapped to the diterpenoid biosynthesis pathway, including SmCYP716A89, SmCYP716D25, SmCYP728D17, SmCYP76A35, SmCYP76S7, SmCYP76AK2, SmCYP76AK3, SmCYP76AH1, SmCYP76G16 and SmCYP701A40. Among them, 2 belonged to the CYP716 family, 1 belonged to the CYP728 family, 6 belonged to the CYP76 family and 1 belonged to the CYP701 family. CYP701 is involved in the biosynthesis of gibberellins, which are diterpenoid acids [37]. These CYP450s are likely to be involved in these terpenoid biosynthesis pathways, and future experiments are required to test this hypothesis.

### Quantification and validation of the expression levels of CYP450 genes

**Figure 7:**
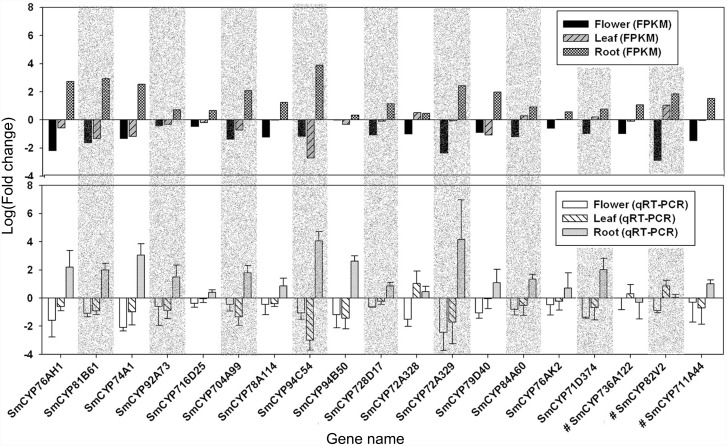
Validation of the expression patterns of 18 randomly selected CYP450 genes and CYP76AH1 across the three tissues of *S. miltiorrhiza*. Fold changes of transcript levels in flower, leaf and root tissues of S. *miltiorrhiza* are shown. The average expression levels in three tissues were arbitrarily set to 1. The error bars represent the standard error relative to the mean (SEM). ‘#’ indicates that the genes did not show similar relative expression levels between RNA-Seq results and qRT-PCR results.

The expression levels (FPKM) of the 116 full-length CYP450 genes were calculated using the Trinity program [30] and are shown in S4 Table. To validate the expression levels of CYP450 genes obtained from the RNA-Seq experiment, we performed qRT-PCR on 18 randomly selected CYP450 genes and SmCYP76AH1. RNAs were extracted from three biological replicates for each tissue type, and each biological replicate had three technical replicates for the qRT-PCR experiments. For each gene, the mean expression level of all biological replicates and technical replicates for each tissue type was calculated. Two methods were used to compare the RNA-Seq and qPCR results. First, the mean expression levels in the three tissue types for each gene were ordered. If the order of expression levels obtained from the RNA-Seq result was the same as that from the qRT-PCR result, the RNA-Seq result was considered to be validated, As shown in Fig. 7, 15 of 18 (78.9%) results were validated. Second, for each gene, the average expression levels for the three biological replicates were calculated. Then, the Pearson correlation coefficients of the tissue expression profiles of each gene obtained by RNA-Seq and qPCR were calculated. The results are shown in S5 Table, and eleven gene pairs had r> = 0.9. These data suggested that the expression patterns deduced from the FPKM values in our transcriptome analyses were reliable and can be used in downstream gene expression analyses.

**Figure 8:**
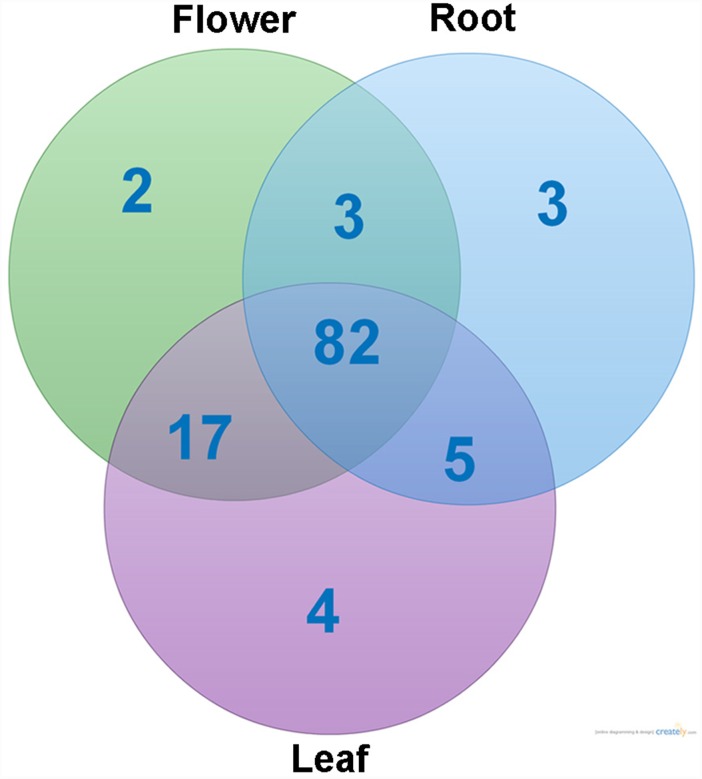
Venn diagram showing the numbers of CYP450 genes expressed across the three tissues.

**Figure 9:**
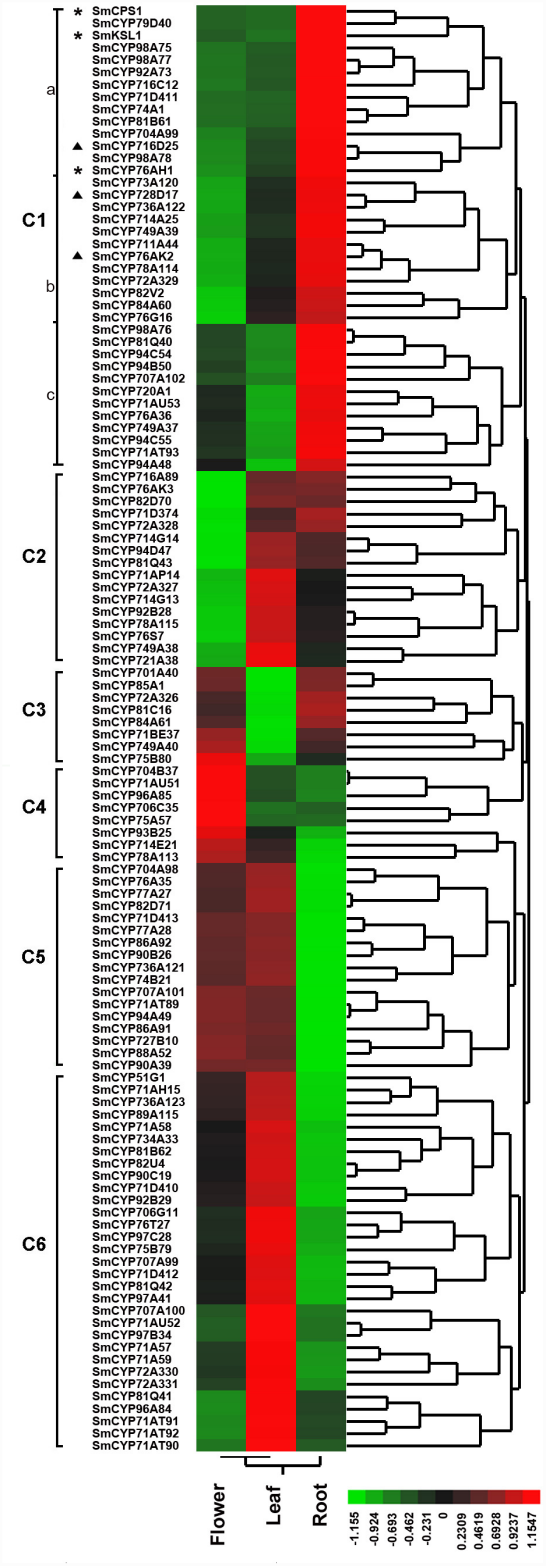
Clustering analyses of the 116 full-length CYP450 genes based on their expression profiles obtained from the RNA-Seq experiment. For data preparation, the expression levels represented by FPKM values were floored, log transformed and mean-centered, as described in the method section. Hierarchical clustering was conducted using JMP software. The heat map reflects the relative expression levels of genes in the three tissues compared to their mean expression levels across the three tissues. The six main clusters, C1–C6, and three sub-clusters, C1a, C1b and C1c, are indicated with square brackets. The color scale corresponds to log (FPKM+1)/(mean expression across the three tissue types). The green color indicates down-regulated expression compared to the mean, and the red color indicates up-regulated expression compared to the mean. The tissues names are shown below the heat map. “*” indicates the reference gene, and “▴” indicates the target genes.

To study the tissue-specific expression of CYP450 genes, we first examined the numbers of genes expressed in the three tissue types. As shown in Fig. 8, a total of 82 CYP450 genes were expressed in all three tissue*s*. Among them, there were 2, 3 and 4 CYP450 genes expressed only in the flower, root and leaf tissues, respectively (Fig. 8). To study the co-expression patterns of CYP450 genes, we performed hierarchical clustering of the expression profiles of these 116 full-length CYP450 genes, SmCPS1, SmKSL1 and SmCYP76AH1 using the Euclidean distance as the metric and Ward’s method. As shown in Fig. 9, six main clusters were readily discernable, which were named C1 to C6. The clusters C1, C4 and C6 possessed the full-length CYP450 genes that showed the highest expression levels in root (37 genes), flower (8 genes) and leaf (31 genes) tissues, respectively. C1 could be further divided into three sub-clusters, C1a, C1b and C1c. C1a had 13 genes, which were expressed similarly in leaf and flower tissues. C1b had 12 genes, which were expressed at the lowest level in the flower tissue. Lastly, C1c had 12 genes, which were expressed at the lowest levels in the leaf tissue. SmCYP76AH1, which is the reference gene involved in tanshinone biosynthesis, together with SmCPS1 and SmKSL1, was found in the cluster C1a and marked with “*” in Fig. 9. Furthermore, the full-length CYP450 genes in clusters C2 (16 genes), C3 (8 genes) and C5 (17 genes) showed the lowest gene expression levels in the flower, leaf and root tissues, respectively. The various expression levels across the different tissue types reflect the unique biological functions of these genes, which need to be elucidated in future studies.

### Identification of candidates CYP450 genes involved in tanshinone biosynthesis

We then identified CYP450 genes that are potentially involved in tanshinone biosynthesis based on the two following criteria: (1) co-expression with a tanshinone biosynthesis marker gene, *CYP76AH1*, which was previously shown to catalyze a unique four-electron oxidation cascade on miltiradiene to produce ferruginol, a step in the diterpenoid biosynthesis pathway in both rhizomes and hairy roots [11], and (2) mapping of the genes to the diterpenoid biosynthesis pathway. The correlation coefficients between the expression profiles of the 115 full-length CYP450 genes and that of SmCYP76AH1 were calculated. Of those belonging to the C1 cluster, 34 CYP450s were found to have correlation coefficients (r) >0.9 (S4 Table). Among these 34 genes, SmCYP76AK2, SmCYP716D25 and SmCYP728D17 were mapped to the diterpenoid biosynthesis pathway by KEGG pathway analyses. SmCYP76AK2 belongs to the CYP76 family, which has undergone significant expansion after speciation (Fig. 4, shaded area). In contrast, SmCYP716D25 and SmCYP728D17 are closely related to SlCYP716 and SmCYP728B22, respectively (Fig. 4, shaded areas). Unfortunately, their functions are currently unknown. Interestingly, all three CYP450 genes were expressed at the highest level in root tissue, where the active compounds were most abundant [38]. Therefore, they are likely to play important roles in tanshinone biosynthesis, and their exact functions require further investigation.

#### Identification of antisense transcripts corresponding to CYP450 genes and correlation analysis of their expression levels

We identified 36 antisense transcripts corresponding to 27 CYP450 genes. Among them, the SmCYP77A28, SmCYP81Q43 and SmCYP89A115 genes each had 3 antisense transcripts, while the SmCYP71A58, SmCYP76G16 and SmCYP81Q40 genes each had 2 antisense transcripts. The remaining 21 genes had 1 antisense transcript. The Pearson correlation coefficients (r) between the expression profiles of antisense transcripts and those of their corresponding CYP450 genes were calculated (S6 Table). Using 0.9 and −0.9 as a cutoff for significant correlation, we found 12 pairs of transcripts that were positive correlated and 3 that were negatively correlated. Whether there are any interactions between these pairs of sense and antisense transcripts remains to be studied.

### Establishment of a web resource for CYP450 genes from *S. miltiorrhiza*

A web resource has been set up for CYP450 genes from *S. miltiorrhiza* (SMCYP450, http://www.herbalgenomics.org/samicyp450). SMCYP450 is a database, as well as a web server, that contains several functional modules. SMCYP450 has integrated information regarding the CYP450 genes of *S. miltiorrhiza* from previous studies and has generated a reference CYP450 gene set, which contains 116 full-length and 135 partial CYP450 genes. A gene page has also been constructed for each gene to describe its information, such as type, clan, family, subfamily, gene sequence, CDS sequence and protein sequence. Furthermore, this resource contains a “search” module that allows users to search the database with their query sequence and a “retrieve” module that allows the search results to be retrieved. A “compare2sequence” module allows the comparison of a user’s CYP450 gene with that from the SMCYP450. Once new CYP450 genes are identified, they will be incorporated into the SMCYP450 database. Ultimately, SMCYP450 might become a standardized resource for CYP450 genes from *S. miltiorrhiza*, expediting the functional characterization of CYP450 genes.

## Discussion

As described earlier, despite several studies focusing on the identification and characterization of CYP450 genes from *S. miltiorrhiza*, information relating to these genes is scattered in the literature, and many of their sequences remain proprietary and not accessible to the general public. A lack of a systematic nomenclature, as well as a centralized resource for CYP450 genes from *S. miltiorrhiza*, has become a bottleneck in research efforts attempting to elucidate the biosynthetic pathways for active compounds in *S. miltiorrhiza*. The current study was initiated to overcome these limitations by (1) identifying and manual curating a large set of CYP450 genes; (2) establishing a standardized nomenclature for these CYP450 genes; (3) mining the sequences to identify those that are likely involved in diterpenoid biosynthesis; and (4) setting up an on-line resource for CYP450 genes from *S. miltiorrhiza.*

### CYP450 genes discovered in this study

In total, we identified 116 full-length and 135 partial CYP450 genes. Among the 116 CYP450 genes, eight genes have been previously characterized. For example, one CYP450, SmCYP73A120, was previously identified as cinnamate 4-hydroxylase (C4H) and three CYP450s, SmCYP98A75, SmCYP98A76 and SmCYP98A77, were previously identified as β-coumaryl-CoA 3′-hydroxylase (CS3′H, ACA64046), β-coumaroyl shikimate 3′-hydroxylase (CS’3H, ACA64047) and β-coumarate 3-hydroxylase (C3H, ACA64048), respectively, which are directly linked to the accumulation of rosmarinic acid (RA) and its derivative, salvianolic acid B (SAB), in *S. miltiorrhiza* [39]. In addition, SmCYP98A78, which is the allelic variant of SmCYP98A14 (accession no. ADP00279), has been shown to be involved in the accumulation of phenolic acids in the hairy root cultures of *S. miltiorrhiza* by introducing the 3-hydroxyl group to form RA [40]. CYP76AH1 catalyzes a unique four-electron oxidation cascade on miltiradiene to produce ferruginol, both in vitro and in vivo [11]. In a previous study, six candidate CYP450 genes (JX422213, JX422214, JX422215, JX422216, JX422217 and JX422218) were found to be co-regulated with the diterpenoid synthase genes in both rhizome and hairy roots [11]. In our study, we found that SmCYP71D411 and SmCYP71D374 were more than 99% similar to two CYP450 genes, ACR57218 and ACR57217, of *S. miltiorrhiza*. While more than 60 CYP450 genes had previously been described, we could not find them in the international DNA and protein sequence databases. As a result, the remaining 108 full-length CYP450 sequences were all considered novel.

### Potential candidate CYP450 genes involved in tanshinone biosynthesis

CYP450 is the largest family of enzymes controlling primary and secondary metabolism and is responsible for the synthesis of diverse compounds such as lignin, pigments, defense compounds, fatty acids, hormones, and signaling molecules involved in plant growth, development and defense [33]. In the terpenoid biosynthesis pathways, CYP450 proteins are involved in the biosynthesis of various classes of compounds [41], which further increase the structural diversity of these terpenoids. Recently, the crucial roles that CYP450 genes play in the biosynthesis of terpenoids in medicinal plants have been reviewed in detail [42]. In total, 52 genes, which belong to 16 families, have been reported to be involved in terpenoid biosynthesis. The families and the numbers of genes in each family are listed below: CYP51 (1 gene), CYP71 (18), CYP72 (2), CYP76 (2), CYP88 (2), CYP93 (3), CYP97 (4), CYP701 (2) CYP705 (1), CYP706 (1), CYP707 (1), CYP714 (1), CYP716 (1), CYP720 (1), CYP725 (6), CYP735 (1) and the unassigned (5).

It might be surprising at first that genes from such a large number of diverse CYP450 families were found to be involved in terpenoid biosynthesis. However, terpenoids are large groups of bioactive compounds found in almost all plant species (Zerbe et al. 2013), and the diversity of terpenoids might result from the diversity of the enzymes involved in their production. Hence, it should be of no surprise that CYP450 genes from various families have been implicated in their biosynthesis. The CYP450 genes must have undergone dramatic diversification in various plant species to fulfill the need for the production of the large number of terpenoids. This also suggests that inferring the involvement of CYP450 genes in terpenoid biosynthesis by sequence similarity alone might be problematic and that experimental validation must be conducted. In our study, we have identified three CYP450 genes that might be involved in terpenoid biosynthesis, based on their sequence similarity and co-expression with marker genes. Among these three genes, SmCYP716D25, SmCYP76AK2 and SmCYP728D17, two belong to the families listed above. The third gene, CYP728, has not been previously linked to terpenoid biosynthesis. Its exact role remains unknown.

### Limitations of current work and future plans

Due to the unavailability of the whole genome sequence of *S. miltiorrhiza*, the current study was not able to identify all CYP450 genes in *S. miltiorrhiza*. For the same reason, we have only identified approximately one third of all CYP450 genes with full-length coding sequences. Consequently, the primary task for future studies is to identify all CYP450 genes in *S. miltiorrhiza* with full-length coding sequences. This task would depend on the completion of the *S. miltiorrhiza* whole genome sequencing project, which is current under way. Alternatively, 5′ RACE and 3′ RACE can be used to obtain the full length sequences for the CYP450 genes identified in the current study. Once the complete reservoir of CYP450 sequences in *S. miltiorrhiza* has been obtained and the full-length sequences have been confirmed, the functional characterization of these CYP450 genes will be the next logical step. This characterization can be done, in theory, either by the in vitro expression of the proteins, followed by activity detection in vitro, or by knock-in and knock-out of the genes of interest, followed by activity detection in vivo, depending on the availability of robust experimental systems for the particular species under study. Ultimately, the CYP450 genes involved in the diterpenoid biosynthesis pathway will likely be identified, laying the foundation for diterpenoid production in vitro.

## Supporting Information

S1 Table **Primers used for qRT-PCR.**
(DOC)

S2 Table **Conserved domains of full-length CYP450s in** ***S. miltiorrhiza*** **.** The conserved domains were predicted using the Pfam HMM model PF00067 with 463 amino acids in length.
(DOC)

S3 Table **Conserved domains of partial CYP450s in** ***S. miltiorrhiza*** **.** The conserved domains were predicted using the Pfam HMM model PF00067 with 463 amino acids in length.
(DOC)

S4 Table **Expression levels (FPKM) of full-length CYP450 genes across three tissues in** ***S. miltiorrhiza*** **and the comparison of their expression profiles to that of the marker gene** ***CYP76AH1.***
(DOC)

S5 Table **Correlation analyses of tissue expression profiles obtained from RNA-Seq and qPCR experiments.**
(XLS)

S6 Table **Correlation analyses of expression profiles for the antisense transcripts and their sense CYP450 genes.**
(XLS)
