# Correction: Computational Identification and Systematic Classification of Novel Cytochrome P450 Genes in *Salvia miltiorrhiza*

**DOI:** 10.1371/journal.pone.0120063

**Published:** 2015-03-25

**Authors:** The PLOS ONE Staff

Table 1 is missing the GenBank Accession numbers for column 7. The authors have provided a corrected Table 1 below.

**Table 1:** List of full-length CYP450s of *S. miltiorrhiza* identified in this study.

No.	Gene Name	Type	CYPClan	CYPFamily	CYPSubfamily	Accession No	Length	PI	Mol. wt (kDa)	Loc^a^
1	SmCYP98A75	A	71	CYP98	CYP98A	KP337735	509	8.96	57.9	S
2	SmCYP73A120	A	71	CYP73	CYP73A	KP337679	504	9.11	57.7	S
3	SmCYP93B25	A	71	CYP93	CYP93B	KP337723	510	8.59	57.4	*
4	SmCYP98A76	A	71	CYP98	CYP98A	KP337736	512	8.55	58.0	S
5	SmCYP98A77	A	71	CYP98	CYP98A	KP337737	508	8.88	57.2	*
6	SmCYP51G1	non-A	51	CYP51	CYP51G	KP337653	492	8.09	55.3	S
7	SmCYP701A40	A	71	CYP701	CYP701A	KP337739	519	7.22	58.8	S
8	SmCYP704A98	non-A	86	CYP704	CYP704A	KP337740	511	6.74	59.2	S
9	SmCYP704B37	non-A	86	CYP704	CYP704B	KP337742	506	7.16	58.2	S
10	SmCYP704A99	non-A	86	CYP704	CYP704A	KP337741	506	6.9	57.7	S
11	SmCYP706C35	A	71	CYP706	CYP706C	KP337743	518	8.17	57.7	*
12	SmCYP706G11	A	71	CYP706	CYP706G	KP337744	507	9.06	56.8	*
13	SmCYP707A99	non-A	85	CYP707	CYP707A	KP337745	488	9.27	55.1	S
14	SmCYP707A100	non-A	85	CYP707	CYP707A	KP337746	481	9.45	54.9	*
15	SmCYP707A101	non-A	85	CYP707	CYP707A	KP337747	479	9.34	54.9	S
16	SmCYP707A102	non-A	85	CYP707	CYP707A	KP337748	470	8.6	53.0	S
17	SmCYP711A44	non-A	711	CYP711	CYP711A	KP337749	514	9.37	57.6	S
18	SmCYP714A25	non-A	72	CYP714	CYP714A	KP337750	527	8.64	59.1	S
19	SmCYP714E21	non-A	72	CYP714	CYP714E	KP337751	523	8.71	58.5	S
20	SmCYP716A89	non-A	85	CYP716	CYP716A	KP337754	479	8.76	54.0	S
21	SmCYP716C12	non-A	85	CYP716	CYP716C	KP337755	476	9	53.4	S
22	SmCYP728D17	non-A	85	CYP728	CYP728D	KP337760	483	8.9	55.4	S
23	SmCYP716D25	non-A	85	CYP716	CYP716D	KP337756	477	9.13	54.3	S
24	SmCYP71AU51	A	71	CYP71	CYP71AU	KP337664	505	6.62	56.4	*
25	SmCYP71AU52	A	71	CYP71	CYP71AU	KP337665	500	6.66	56.4	*
26	SmCYP71AH15	A	71	CYP71	CYP71AH	KP337657	496	6.05	55.5	S
27	SmCYP71AP14	A	71	CYP71	CYP71AP	KP337658	522	6.14	58.7	*
28	SmCYP71A57	A	71	CYP71	CYP71A	KP337654	497	8.47	55.8	*
29	SmCYP71A58	A	71	CYP71	CYP71A	KP337655	505	6.33	56.9	S
30	SmCYP71A59	A	71	CYP71	CYP71A	KP337656	500	6.27	56.0	*
31	SmCYP71D410	A	71	CYP71	CYP71D	KP337669	504	7.65	56.5	S
32	SmCYP71D411	A	71	CYP71	CYP71D	KP337670	498	7.2	56.6	S
33	SmCYP71D374	A	71	CYP71	CYP71D	KP337668	502	8.73	57.1	*
34	SmCYP71BE37	A	71	CYP71	CYP71BE	KP337667	498	8.34	56.8	*
35	SmCYP71D412	A	71	CYP71	CYP71D	KP337671	502	6.7	57.2	*
36	SmCYP71D413	A	71	CYP71	CYP71D	KP337672	511	6.14	58.2	*
37	SmCYP720A1	non-A	85	CYP720	CYP720A	KP337757	494	8.97	56.5	S
38	SmCYP72A326	non-A	72	CYP72	CYP72A	KP337673	518	9.3	59.1	S
39	SmCYP72A327	non-A	72	CYP72	CYP72A	KP337674	518	9.04	59.1	S
40	SmCYP72A328	non-A	72	CYP72	CYP72A	KP337675	517	9.01	59.6	S
41	SmCYP72A329	non-A	72	CYP72	CYP72A	KP337676	512	9.03	58.1	S
42	SmCYP72A330	non-A	72	CYP72	CYP72A	KP337677	517	9.1	59.1	S
43	SmCYP72A331	non-A	72	CYP72	CYP72A	KP337678	516	8.72	59.0	S
44	SmCYP734A33	non-A	72	CYP734	CYP734A	KP337761	522	9.1	59.6	S
45	SmCYP749A37	non-A	72	CYP749	CYP749A	KP37765	508	8.95	58.3	S
46	SmCYP749A38	non-A	72	CYP749	CYP749A	KP337766	508	9.25	58.4	S
47	SmCYP721A38	non-A	72	CYP721	CYP721A	KP337758	506	8.93	58.3	S
48	SmCYP749A39	non-A	72	CYP749	CYP749A	KP337767	511	9.14	58.5	S
49	SmCYP749A40	non-A	72	CYP749	CYP749A	KP337768	509	9	57.9	S
50	SmCYP727B10	non-A	727	CYP727	CYP727B	KP337759	502	8.59	56.4	S
51	SmCYP714G13	non-A	72	CYP714	CYP714G	KP337752	506	9.34	56.4	S
52	SmCYP714G14	non-A	72	CYP714	CYP714G	KP337753	514	9.16	57.5	S
53	SmCYP736A121	A	71	CYP736	CYP736A	KP337762	496	6.72	56.4	*
54	SmCYP736A122	A	71	CYP736	CYP736A	KP337763	502	6.82	57.1	*
55	SmCYP736A123	A	71	CYP736	CYP736A	KP337764	488	7.07	55.3	*
56	SmCYP74A1	non-A	74	CYP74	CYP74A	KP337680	519	8.88	58.0	C
57	SmCYP74B21	non-A	74	CYP74	CYP74B	KP337681	483	5.91	53.9	*
58	SmCYP75B79	A	71	CYP75	CYP75B	KP337683	511	7.73	56.2	*
59	SmCYP75B80	A	71	CYP75	CYP75B	KP337684	514	7.3	56.5	*
60	SmCYP92B28	A	71	CYP92	CYP92B	KP337721	501	6.55	56.5	S
61	SmCYP75A57	A	71	CYP75	CYP75A	KP337682	516	8.43	57.6	S
62	SmCYP92A73	A	71	CYP92	CYP92A	KP337720	509	8.93	57.9	*
63	SmCYP92B29	A	71	CYP92	CYP92B	KP337722	509	6.69	57.7	*
64	SmCYP76AH1	A	71	CYP76	CYP76AH	KP337687	495	7.73	55.5	*
65	SmCYP76AK2	A	71	CYP76	CYP76AK	KP337688	494	8.72	55.8	*
66	SmCYP76S7	A	71	CYP76	CYP76S	KP337691	494	7.67	55.8	*
67	SmCYP76AK3	A	71	CYP76	CYP76AK	KP337689	491	8.85	55.4	*
68	SmCYP76T27	A	71	CYP76	CYP76T	KP337692	486	8.83	55.0	*
69	SmCYP76A35	A	71	CYP76	CYP76A	KP337685	503	8.25	57.8	*
70	SmCYP76A36	A	71	CYP76	CYP76A	KP337686	513	8.9	57.7	*
71	SmCYP77A27	A	71	CYP77	CYP77A	KP337693	512	9.13	57.6	S
72	SmCYP77A28	A	71	CYP77	CYP77A	KP337694	505	9.16	56.8	*
73	SmCYP78A113	A	71	CYP78	CYP78A	KP337695	511	8.61	57.3	S
74	SmCYP78A114	A	71	CYP78	CYP78A	KP337696	533	9.24	59.1	*
75	SmCYP78A115	A	71	CYP78	CYP78A	KP337697	526	9.1	57.9	S
76	SmCYP79D40	A	71	CYP79	CYP79D	KP337698	522	8.98	58.7	S
77	SmCYP81Q40	A	71	CYP81	CYP81Q	KP337702	493	7.3	55.8	*
78	SmCYP81B61	A	71	CYP81	CYP81B	KP337699	510	8.9	58.1	S
79	SmCYP81B62	A	71	CYP81	CYP81B	KP337700	498	7.68	55.9	S
80	SmCYP81Q41	A	71	CYP81	CYP81Q	KP337703	494	7.68	56.0	*
81	SmCYP81Q42	A	71	CYP81	CYP81Q	KP337704	493	6.27	56.1	*
82	SmCYP81Q43	A	71	CYP81	CYP81Q	KP337705	494	7.68	56.0	*
83	SmCYP81C16	A	71	CYP81	CYP81C	KP337701	498	8.07	55.8	S
84	SmCYP82V2	A	71	CYP82	CYP82V	KP337709	534	6.61	60.2	S
85	SmCYP82D70	A	71	CYP82	CYP82D	KP337706	516	8.9	57.6	*
86	SmCYP82D71	A	71	CYP82	CYP82D	KP337707	516	6.59	57.4	S
87	SmCYP82U4	A	71	CYP82	CYP82U	KP337708	522	7.71	58.8	S
88	SmCYP71AT89	A	71	CYP71	CYP71AT	KP337659	499	8.87	56.1	*
89	SmCYP71AT90	A	71	CYP71	CYP71AT	KP337660	496	7.69	55.6	*
90	SmCYP71AT91	A	71	CYP71	CYP71AT	KP337661	506	8.44	56.7	*
91	SmCYP71AT92	A	71	CYP71	CYP71AT	KP337662	506	6.15	57.4	S
92	SmCYP71AT93	A	71	CYP71	CYP71AT	KP337663	491	9.05	55.3	*
93	SmCYP84A60	A	71	CYP84	CYP84A	KP337710	517	5.68	58.0	S
94	SmCYP84A61	A	71	CYP84	CYP84A	KP337711	517	6.61	58.0	S
95	SmCYP85A1	non-A	85	CYP85	CYP85A	KP337712	463	8.88	53.3	S
96	SmCYP86A91	non-A	86	CYP86	CYP86A	KP337713	555	8.48	62.3	S
97	SmCYP86A92	non-A	86	CYP86	CYP86A	KP337714	525	8.28	59.6	S
98	SmCYP96A84	non-A	86	CYP96	CYP96A	KP337730	485	8.96	55.0	S
99	SmCYP96A85	non-A	86	CYP96	CYP96A	KP337731	502	8.42	57.8	*
100	SmCYP88A52	non-A	85	CYP88	CYP88A	KP337715	487	9.26	56.2	S
101	SmCYP89A115	A	71	CYP89	CYP89A	KP337716	500	7.66	57.7	S
102	SmCYP90A39	non-A	85	CYP90	CYP90A	KP337717	478	9.12	54.4	*
103	SmCYP90B26	non-A	85	CYP90	CYP90B	KP337718	476	9.05	53.9	S
104	SmCYP90C19	non-A	85	CYP90	CYP90C	KP337719	488	8.4	55.5	S
105	SmCYP94A48	non-A	86	CYP94	CYP94A	KP337724	502	8.85	57.7	S
106	SmCYP94A49	non-A	86	CYP94	CYP94A	KP337725	502	8.6	57.1	S
107	SmCYP94B50	non-A	86	CYP94	CYP94B	KP337726	505	8.89	56.1	S
108	SmCYP94C54	non-A	86	CYP94	CYP94C	KP337727	491	7.63	55.1	S
109	SmCYP94C55	non-A	86	CYP94	CYP94C	KP337728	488	8.45	56.2	S
110	SmCYP94D47	non-A	86	CYP94	CYP94D	KP337729	501	9.02	57.1	*
111	SmCYP97A41	non-A	97	CYP97	CYP97A	KP337732	612	5.89	68.6	C
112	SmCYP97B34	non-A	97	CYP97	CYP97B	KP337733	582	6.31	65.0	C
113	SmCYP97C28	non-A	97	CYP97	CYP97C	KP337734	542	6.12	60.1	C
114	SmCYP98A78	A	71	CYP98	CYP98A	KP337738	508	8.48	57.6	S
115	SmCYP76G16	A	71	CYP76	CYP76G	KP337690	508	8.5	57.0	*
116	SmCYP71AU53	A	71	CYP71	CYP71AU	KP337666	498	9.37	56.6	*

## References

[1] Chen H, Wu B, Nelson DR, Wu K, Liu C (2014) Computational Identification and Systematic Classification of Novel Cytochrome P450 Genes in *Salvia miltiorrhiza* . PLoS ONE 9(12): e115149 doi: 10.1371/journal.pone.0115149 25493946 10.1371/journal.pone.0115149 PMC4262458

