# Supplementary material for: Computational Identification and Systematic Classification of Novel Cytochrome P450 Genes in Salvia miltiorrhiza
Source: PLoS One. 2014 Dec 10;9(12):e115149. doi: 10.1371/journal.pone.0115149 (PMC4262458; doi:10.1371/journal.pone.0115149)
Supplement: S1 Table — Primers used for qRT-PCR. (DOC) [file pone.0115149.s001.doc]

**Table S1 Primers used for q**RT-PCR.

| Gene name | Forward Primer | Reverse Primer |
| --- | --- | --- |
| SmCYP81B61 | ACATGGCGTTACGTGTCATAG | GCCATATCGACCATCTCCTTATC |
| SmCYP82V2 | GGCGACTCTATTGAACCTCATC | GCATTGCTCGAGTGTTGTTTAG |
| SmCYP74A1 | CCACCTACGTCCAGATGTTTC | GGATCGGGCTAGGAAGTTAAAG |
| SmCYP92A73 | CACTCACCTTTCTCTCACTACAC | AGCCGATGAGGTTGAGATTG |
| SmCYP736A122 | CAACATCGTCTTCTCTCCCTAC | CATGTTCTCCTCCTCCCTTATG |
| SmCYP716D25 | GAGGAGGATGGTGTCGTATTTC | ATGCTGCTGTGAGACCATATC |
| SmCYP704A99 | GCAGCTAAACTTGCCCATTTAC | CACAGAGTCCAGTGAGGATTTC |
| SmCYP78A114 | CTGAGAGATTTGGTGGAGGAAG | TACGTCGAAATCCGCTAAGAAG |
| SmCYP94C54 | GGGCCCACTAGATTAGATCATTAC | GGAGACGAAGACGCCTAATAAC |
| SmCYP76AK2 | CCTCTCCCAAGGAAATGAGTATG | CCGATCCTGCGATGATTATGT |
| SmCYP71D374 | TTCTCCGCCTACAACGAGTA | CTGATGTGGCCGAAGGATTT |
| SmCYP94B50 | CTCCACTCCATTCACTTTCCTC | GAGGAGGCAGAAGATGAGAAAC |
| SmCYP728D17 | CGGCAAGTCCTTCGAGATATT | GGTATATGGATCCGTCCAACTG |
| SmCYP72A328 | GGTAGCGCAGACGACTTATT | CTTGCACTCCTCGATCACTT |
| SmCYP711A44 | GGCTTTGACTAATCCGTTGTTTC | AGCATCTCCAGCACATCATC |
| SmCYP72A329 | ACGAGGAAGGAACCAAGATATTC | CTCCTTCAACCTCCGGTTATTC |
| SmCYP79D40 | ACCGAGTTGTAGGCAAGAATAG | AAAGGTGCCAGTGGATGTAG |
| SmCYP84A60 | GGTTTCGGAGTCTGAGGATTT | TCTTCTGGGCTTCGCATTAG |
| SmCYP76AH1 | CAATGCGCCAAAGAAGGATG | AGCATGAGATGGGTGAAGTG |
| SmActin | TGTCAGCAACTGGGATGATATG | CGATTGGCCTTGGGATTAAGA |
