# Supplementary material for: Computational Identification and Systematic Classification of Novel Cytochrome P450 Genes in Salvia miltiorrhiza
Source: PLoS One. 2014 Dec 10;9(12):e115149. doi: 10.1371/journal.pone.0115149 (PMC4262458; doi:10.1371/journal.pone.0115149)
Supplement: S2 Table — Conserved domains of full-length CYP450s in S. miltiorrhiza . The conserved domains were predicted using the Pfam HMM model PF00067 with 463 amino acids in length. (DOC) [file pone.0115149.s002.doc]

**Table S2 Conserved domains of full-length CYP450s in *S. miltiorrhiza*. The conserved domains were predicted using the PFAM HMM model PF00067 with 463 amino acids in length.**

| No. | CYP450 Gene | Alignment | | Envelope | | hmm | | Bit Score | E-value |
| --- | --- | --- | --- | --- | --- | --- | --- | --- | --- |
|  |  | Start | End | Start | End | Start | End |  |  |
| 1 | SmCYP98A75 | 29 | 477 | 29 | 488 | 1 | 450 | 345.7 | 3.20E-103 |
| 2 | SmCYP73A120 | 34 | 494 | 34 | 498 | 1 | 459 | 383.2 | 1.30E-114 |
| 3 | SmCYP93B25 | 33 | 484 | 32 | 491 | 2 | 455 | 347.9 | 7.10E-104 |
| 4 | SmCYP98A76 | 32 | 484 | 32 | 492 | 1 | 452 | 342.9 | 2.30E-102 |
| 5 | SmCYP98A77 | 29 | 486 | 29 | 490 | 1 | 458 | 338.7 | 4.30E-101 |
| 6 | SmCYP51G1 | 44 | 462 | 43 | 482 | 2 | 439 | 225 | 1.30E-66 |
| 7 | SmCYP701A40 | 48 | 492 | 46 | 505 | 3 | 447 | 236.1 | 5.40E-70 |
| 8 | SmCYP704A98 | 52 | 494 | 40 | 499 | 18 | 455 | 258.3 | 1.00E-76 |
| 9 | SmCYP704B37 | 27 | 500 | 25 | 502 | 3 | 461 | 233.6 | 3.30E-69 |
| 10 | SmCYP704A99 | 37 | 481 | 32 | 489 | 7 | 454 | 242.6 | 5.90E-72 |
| 11 | SmCYP706C35 | 50 | 502 | 50 | 506 | 1 | 459 | 347.9 | 6.80E-104 |
| 12 | SmCYP706G11 | 32 | 492 | 32 | 495 | 1 | 460 | 348.4 | 4.90E-104 |
| 13 | SmCYP707A99 | 36 | 447 | 36 | 457 | 1 | 438 | 245.1 | 1.00E-72 |
| 14 | SmCYP707A100 | 35 | 445 | 35 | 456 | 1 | 442 | 239.9 | 3.80E-71 |
| 15 | SmCYP707A101 | 41 | 451 | 41 | 461 | 1 | 441 | 248.5 | 9.80E-74 |
| 16 | SmCYP707A102 | 38 | 441 | 38 | 461 | 1 | 438 | 259 | 6.30E-77 |
| 17 | SmCYP711A44 | 36 | 494 | 35 | 504 | 2 | 447 | 279.3 | 4.30E-83 |
| 18 | SmCYP714A25 | 100 | 502 | 92 | 520 | 21 | 441 | 241.4 | 1.40E-71 |
| 19 | SmCYP714E21 | 92 | 501 | 86 | 519 | 26 | 442 | 266.5 | 3.30E-79 |
| 20 | SmCYP716A89 | 35 | 451 | 35 | 469 | 1 | 437 | 215.9 | 7.50E-64 |
| 21 | SmCYP716C12 | 31 | 456 | 31 | 473 | 1 | 443 | 200.7 | 3.10E-59 |
| 22 | SmCYP728D17 | 30 | 461 | 30 | 473 | 1 | 450 | 239.1 | 6.70E-71 |
| 23 | SmCYP716D25 | 33 | 455 | 33 | 470 | 1 | 442 | 216.3 | 5.50E-64 |
| 24 | SmCYP71AU51 | 45 | 485 | 45 | 499 | 1 | 450 | 338.2 | 5.80E-101 |
| 25 | SmCYP71AU52 | 35 | 480 | 35 | 489 | 1 | 450 | 355.9 | 2.60E-106 |
| 26 | SmCYP71AH15 | 35 | 483 | 35 | 489 | 1 | 456 | 308.8 | 4.90E-92 |
| 27 | SmCYP71AP14 | 50 | 504 | 49 | 509 | 2 | 458 | 353 | 2.00E-105 |
| 28 | SmCYP71A57 | 36 | 477 | 36 | 490 | 1 | 450 | 349.7 | 1.90E-104 |
| 29 | SmCYP71A58 | 49 | 490 | 48 | 497 | 1 | 453 | 345.6 | 3.30E-103 |
| 30 | SmCYP71A59 | 35 | 491 | 35 | 495 | 1 | 459 | 335.4 | 4.10E-100 |
| 31 | SmCYP71D410 | 39 | 480 | 38 | 492 | 2 | 452 | 328.3 | 5.90E-98 |
| 32 | SmCYP71D411 | 38 | 468 | 37 | 488 | 2 | 445 | 324.7 | 7.60E-97 |
| 33 | SmCYP71D374 | 40 | 487 | 40 | 494 | 1 | 456 | 336.4 | 2.10E-100 |
| 34 | SmCYP71BE37 | 35 | 482 | 35 | 486 | 1 | 459 | 326.3 | 2.40E-97 |
| 35 | SmCYP71D412 | 24 | 477 | 24 | 491 | 1 | 448 | 339.7 | 2.20E-101 |
| 36 | SmCYP71D413 | 35 | 482 | 35 | 496 | 1 | 450 | 332.9 | 2.50E-99 |
| 37 | SmCYP720A1 | 44 | 464 | 44 | 480 | 1 | 445 | 210.7 | 2.80E-62 |
| 38 | SmCYP72A326 | 89 | 494 | 73 | 513 | 30 | 440 | 264.7 | 1.10E-78 |
| 39 | SmCYP72A327 | 85 | 494 | 75 | 511 | 26 | 440 | 289.9 | 2.70E-86 |
| 40 | SmCYP72A328 | 87 | 490 | 73 | 510 | 30 | 439 | 274.6 | 1.20E-81 |
| 41 | SmCYP72A329 | 84 | 487 | 81 | 504 | 26 | 439 | 276.8 | 2.60E-82 |
| 42 | SmCYP72A330 | 84 | 493 | 72 | 512 | 26 | 440 | 290 | 2.40E-86 |
| 43 | SmCYP72A331 | 91 | 490 | 84 | 505 | 32 | 439 | 266 | 4.70E-79 |
| 44 | SmCYP734A33 | 84 | 503 | 77 | 517 | 23 | 445 | 294.3 | 1.30E-87 |
| 45 | SmCYP749A37 | 86 | 504 | 75 | 505 | 26 | 462 | 230.1 | 3.80E-68 |
| 46 | SmCYP749A38 | 79 | 481 | 68 | 497 | 25 | 439 | 260 | 3.20E-77 |
| 47 | SmCYP721A38 | 82 | 500 | 67 | 501 | 26 | 462 | 290 | 2.40E-86 |
| 48 | SmCYP749A39 | 87 | 486 | 83 | 504 | 31 | 439 | 265 | 9.40E-79 |
| 49 | SmCYP749A40 | 84 | 487 | 75 | 506 | 31 | 443 | 265.3 | 7.60E-79 |
| 50 | SmCYP727B10 | 43 | 485 | 41 | 496 | 3 | 451 | 163.1 | 7.80E-48 |
| 51 | SmCYP714G13 | 74 | 496 | 39 | 503 | 21 | 456 | 275.3 | 7.30E-82 |
| 52 | SmCYP714G14 | 88 | 490 | 79 | 509 | 24 | 440 | 258.1 | 1.20E-76 |
| 53 | SmCYP736A121 | 31 | 477 | 31 | 488 | 1 | 451 | 335.9 | 3.10E-100 |
| 54 | SmCYP736A122 | 34 | 476 | 34 | 493 | 1 | 447 | 341.3 | 7.00E-102 |
| 55 | SmCYP736A123 | 27 | 473 | 27 | 479 | 1 | 457 | 338 | 6.90E-101 |
| 56 | SmCYP74A1 | 349 | 450 | 345 | 496 | 298 | 399 | 47.4 | 9.70E-13 |
| 57 | SmCYP74B21 | 314 | 425 | 304 | 433 | 292 | 403 | 63.3 | 1.40E-17 |
| 58 | SmCYP75B79 | 33 | 488 | 33 | 498 | 1 | 452 | 363.9 | 9.30E-109 |
| 59 | SmCYP75B80 | 29 | 476 | 29 | 498 | 1 | 441 | 341.9 | 4.50E-102 |
| 60 | SmCYP92B28 | 32 | 485 | 32 | 491 | 1 | 457 | 310.5 | 1.50E-92 |
| 61 | SmCYP75A57 | 41 | 495 | 41 | 501 | 1 | 455 | 381.3 | 5.20E-114 |
| 62 | SmCYP92A73 | 34 | 486 | 34 | 494 | 1 | 455 | 331.7 | 5.50E-99 |
| 63 | SmCYP92B29 | 36 | 494 | 36 | 499 | 1 | 458 | 298.7 | 5.90E-89 |
| 64 | SmCYP76AH1 | 29 | 467 | 29 | 484 | 1 | 443 | 349.9 | 1.70E-104 |
| 65 | SmCYP76AK2 | 33 | 480 | 33 | 487 | 1 | 454 | 327.3 | 1.30E-97 |
| 66 | SmCYP76S7 | 34 | 479 | 34 | 487 | 1 | 454 | 347.4 | 9.70E-104 |
| 67 | SmCYP76AK3 | 30 | 481 | 30 | 486 | 1 | 457 | 347 | 1.30E-103 |
| 68 | SmCYP76T27 | 32 | 471 | 31 | 485 | 2 | 449 | 317 | 1.70E-94 |
| 69 | SmCYP76A35 | 34 | 482 | 34 | 494 | 1 | 450 | 361 | 7.30E-108 |
| 70 | SmCYP76A36 | 34 | 496 | 34 | 504 | 1 | 452 | 332.7 | 2.80E-99 |
| 71 | SmCYP77A27 | 43 | 500 | 43 | 509 | 1 | 454 | 360.6 | 9.50E-108 |
| 72 | SmCYP77A28 | 38 | 493 | 38 | 501 | 1 | 454 | 353.3 | 1.60E-105 |
| 73 | SmCYP78A113 | 56 | 493 | 55 | 504 | 2 | 450 | 266.1 | 4.30E-79 |
| 74 | SmCYP78A114 | 69 | 494 | 68 | 499 | 2 | 436 | 242.2 | 8.00E-72 |
| 75 | SmCYP78A115 | 68 | 494 | 67 | 508 | 2 | 442 | 247.7 | 1.60E-73 |
| 76 | SmCYP79D40 | 41 | 495 | 41 | 511 | 1 | 446 | 275.9 | 4.80E-82 |
| 77 | SmCYP81Q40 | 31 | 472 | 30 | 477 | 2 | 455 | 299.3 | 3.70E-89 |
| 78 | SmCYP81B61 | 35 | 490 | 34 | 495 | 1 | 457 | 323.4 | 1.90E-96 |
| 79 | SmCYP81B62 | 31 | 476 | 30 | 483 | 2 | 455 | 307.2 | 1.50E-91 |
| 80 | SmCYP81Q41 | 32 | 471 | 31 | 477 | 2 | 455 | 290.6 | 1.60E-86 |
| 81 | SmCYP81Q42 | 31 | 474 | 31 | 480 | 1 | 456 | 316.9 | 1.70E-94 |
| 82 | SmCYP81Q43 | 31 | 468 | 30 | 477 | 1 | 452 | 325.3 | 4.80E-97 |
| 83 | SmCYP81C16 | 33 | 465 | 33 | 482 | 1 | 437 | 312.1 | 4.90E-93 |
| 84 | SmCYP82V2 | 44 | 515 | 44 | 520 | 1 | 456 | 302 | 5.60E-90 |
| 85 | SmCYP82D70 | 62 | 497 | 42 | 507 | 19 | 453 | 281.2 | 1.10E-83 |
| 86 | SmCYP82D71 | 38 | 489 | 35 | 501 | 3 | 449 | 316.4 | 2.50E-94 |
| 87 | SmCYP82U4 | 37 | 503 | 36 | 506 | 1 | 457 | 313.2 | 2.30E-93 |
| 88 | SmCYP71AT89 | 32 | 489 | 32 | 493 | 1 | 459 | 361.5 | 5.20E-108 |
| 89 | SmCYP71AT90 | 34 | 481 | 32 | 483 | 3 | 461 | 301.9 | 6.30E-90 |
| 90 | SmCYP71AT91 | 31 | 488 | 31 | 492 | 1 | 459 | 352.1 | 3.70E-105 |
| 91 | SmCYP71AT92 | 32 | 498 | 32 | 500 | 1 | 461 | 348.1 | 6.10E-104 |
| 92 | SmCYP71AT93 | 30 | 481 | 30 | 485 | 1 | 459 | 311.7 | 6.50E-93 |
| 93 | SmCYP84A60 | 41 | 499 | 41 | 505 | 1 | 457 | 328.4 | 5.70E-98 |
| 94 | SmCYP84A61 | 40 | 501 | 40 | 506 | 1 | 458 | 324.7 | 7.60E-97 |
| 95 | SmCYP85A1 | 35 | 443 | 35 | 456 | 1 | 442 | 193.4 | 4.90E-57 |
| 96 | SmCYP86A91 | 28 | 500 | 27 | 508 | 2 | 453 | 212.3 | 9.00E-63 |
| 97 | SmCYP86A92 | 30 | 497 | 30 | 506 | 1 | 452 | 216.2 | 6.1E-64 |
| 98 | SmCYP96A84 | 28 | 462 | 24 | 478 | 6 | 445 | 193.8 | 3.70E-57 |
| 99 | SmCYP96A85 | 29 | 488 | 29 | 489 | 1 | 462 | 193.2 | 5.70E-57 |
| 100 | SmCYP88A52 | 40 | 457 | 40 | 473 | 1 | 437 | 214.1 | 2.60E-63 |
| 101 | SmCYP89A115 | 32 | 491 | 32 | 497 | 1 | 457 | 320.9 | 1.00E-95 |
| 102 | SmCYP90A39 | 30 | 131 | 30 | 146 | 1 | 103 | 22.2 | 4.30E-05 |
| 102 | SmCYP90A39 | 203 | 450 | 153 | 466 | 194 | 444 | 196.2 | 7.30E-58 |
| 103 | SmCYP90B26 | 34 | 452 | 34 | 462 | 1 | 441 | 203.9 | 3.20E-60 |
| 104 | SmCYP90C19 | 44 | 463 | 44 | 483 | 1 | 439 | 186.6 | 5.70E-55 |
| 105 | SmCYP94A48 | 79 | 477 | 35 | 491 | 43 | 440 | 213.8 | 3.20E-63 |
| 106 | SmCYP94A49 | 76 | 476 | 34 | 493 | 42 | 443 | 223.6 | 3.60E-66 |
| 107 | SmCYP94B50 | 41 | 476 | 35 | 492 | 8 | 439 | 207.2 | 3.10E-61 |
| 108 | SmCYP94C54 | 79 | 471 | 56 | 484 | 45 | 449 | 198.9 | 1.00E-58 |
| 109 | SmCYP94C55 | 82 | 465 | 57 | 477 | 47 | 444 | 174.8 | 2.10E-51 |
| 110 | SmCYP94D47 | 30 | 486 | 30 | 496 | 1 | 453 | 226.2 | 5.60E-67 |
| 111 | SmCYP97A41 | 139 | 561 | 124 | 573 | 26 | 451 | 295.9 | 4.20E-88 |
| 112 | SmCYP97B34 | 101 | 571 | 92 | 573 | 23 | 461 | 272.2 | 6.30E-81 |
| 113 | SmCYP97C28 | 92 | 514 | 81 | 527 | 26 | 447 | 275.4 | 6.80E-82 |
| 114 | SmCYP98A78 | 29 | 471 | 29 | 487 | 1 | 446 | 331.7 | 5.60E-99 |
| 115 | SmCYP76G16 | 30 | 475 | 30 | 494 | 1 | 441 | 355.9 | 2.50E-106 |
| 116 | SmCYP71AU53 | 37 | 485 | 37 | 492 | 1 | 455 | 351 | 8.00E-105 |
