# Supplementary material for: Computational Identification and Systematic Classification of Novel Cytochrome P450 Genes in Salvia miltiorrhiza
Source: PLoS One. 2014 Dec 10;9(12):e115149. doi: 10.1371/journal.pone.0115149 (PMC4262458; doi:10.1371/journal.pone.0115149)
Supplement: S3 Table — Conserved domains of partial CYP450s in S. miltiorrhiza . The conserved domains were predicted using the Pfam HMM model PF00067 with 463 amino acids in length. (DOC) [file pone.0115149.s003.doc]

**Table S3 Conserved domains of partial CYP450 in *S. miltiorrhiza*. The conserved domains were predicted using the PFAM HMM model PF00067 with 463 amino acids in length.**

| No | Transcript ID | Alignment | | Envelope | | hmm | | Bit Score | E-value |
| --- | --- | --- | --- | --- | --- | --- | --- | --- | --- |
|  |  | Start | End | Start | End | Start | End |  |  |
| 1 | comp17744_c0_seq1 | 1 | 45 | 1 | 57 | 405 | 449 | 36.3 | 2.3E-09 |
| 2 | comp18093_c0_seq1 | 7 | 107 | 2 | 116 | 343 | 438 | 87.1 | 8.5E-25 |
| 3 | comp23392_c0_seq1 | 25 | 86 | 25 | 90 | 1 | 64 | 44.9 | 5.7E-12 |
| 4 | comp28141_c0_seq1 | 36 | 244 | 36 | 248 | 1 | 215 | 89.8 | 1.3E-25 |
| 5 | comp28658_c0_seq1 | 13 | 117 | 1 | 129 | 80 | 184 | 47.3 | 1.1E-12 |
| 6 | comp28863_c0_seq1 | 2 | 113 | 1 | 115 | 193 | 309 | 61.7 | 4.3E-17 |
| 7 | comp29743_c0_seq1 | 2 | 145 | 1 | 158 | 300 | 447 | 167.6 | 3.3E-49 |
| 8 | comp29827_c0_seq2 | 46 | 464 | 46 | 467 | 1 | 442 | 214.4 | 2.1E-63 |
| 9 | comp31649_c0_seq1 | 1 | 128 | 1 | 129 | 261 | 389 | 123.8 | 6.3E-36 |
| 10 | comp31659_c0_seq1 | 28 | 72 | 21 | 75 | 303 | 347 | 37.4 | 1E-09 |
| 11 | comp33117_c0_seq1 | 4 | 74 | 1 | 74 | 309 | 379 | 83.9 | 8.1E-24 |
| 12 | comp33145_c0_seq1 | 6 | 50 | 2 | 66 | 397 | 441 | 53 | 1.9E-14 |
| 13 | comp33355_c0_seq1 | 42 | 109 | 42 | 111 | 1 | 70 | 33.8 | 1.3E-08 |
| 14 | comp33501_c0_seq1 | 3 | 89 | 1 | 92 | 44 | 134 | 36.8 | 1.6E-09 |
| 15 | comp34309_c0_seq1 | 42 | 98 | 42 | 100 | 1 | 59 | 31.8 | 5.2E-08 |
| 16 | comp35082_c0_seq1 | 29 | 96 | 28 | 96 | 2 | 69 | 35.3 | 4.7E-09 |
| 17 | comp35236_c0_seq1 | 1 | 91 | 1 | 91 | 316 | 407 | 108.7 | 2.5E-31 |
| 18 | comp35236_c1_seq1 | 2 | 47 | 1 | 60 | 402 | 450 | 45 | 5.3E-12 |
| 19 | comp35707_c0_seq1 | 1 | 87 | 1 | 93 | 373 | 457 | 83.6 | 1E-23 |
| 20 | comp36284_c0_seq1 | 3 | 121 | 1 | 123 | 327 | 446 | 146.5 | 8.2E-43 |
| 21 | comp36632_c0_seq1 | 33 | 87 | 33 | 87 | 1 | 57 | 41.2 | 7.5E-11 |
| 22 | comp37470_c0_seq1 | 2 | 151 | 1 | 152 | 174 | 325 | 82.8 | 1.7E-23 |
| 23 | comp37867_c0_seq1 | 1 | 86 | 1 | 88 | 378 | 461 | 68.6 | 3.7E-19 |
| 24 | comp38196_c0_seq1 | 5 | 96 | 1 | 114 | 342 | 437 | 92.6 | 1.9E-26 |
| 25 | comp38958_c0_seq1 | 1 | 52 | 1 | 57 | 405 | 458 | 28.5 | 5.2E-07 |
| 26 | comp39585_c0_seq1 | 1 | 71 | 1 | 76 | 324 | 394 | 96 | 1.8E-27 |
| 27 | comp39795_c0_seq2 | 35 | 404 | 34 | 405 | 1 | 380 | 217.6 | 2.2E-64 |
| 28 | comp40114_c0_seq1 | 19 | 295 | 19 | 299 | 1 | 299 | 95 | 3.4E-27 |
| 29 | comp41037_c0_seq1 | 1 | 76 | 1 | 76 | 281 | 356 | 89.2 | 2E-25 |
| 30 | comp41443_c0_seq1 | 3 | 94 | 1 | 100 | 348 | 438 | 103.2 | 1.2E-29 |
| 31 | comp4234_c0_seq1 | 2 | 95 | 1 | 96 | 30 | 126 | 36.4 | 2.1E-09 |
| 32 | comp43216_c0_seq1 | 2 | 99 | 1 | 100 | 321 | 421 | 115.8 | 1.8E-33 |
| 33 | comp43450_c0_seq1 | 5 | 104 | 2 | 104 | 191 | 290 | 52.7 | 2.4E-14 |
| 34 | comp43518_c0_seq1 | 8 | 193 | 1 | 194 | 134 | 325 | 95.4 | 2.6E-27 |
| 35 | comp43518_c1_seq1 | 46 | 170 | 46 | 170 | 1 | 127 | 67.9 | 5.8E-19 |
| 36 | comp43644_c0_seq1 | 5 | 70 | 2 | 71 | 240 | 303 | 47.1 | 1.2E-12 |
| 37 | comp43811_c0_seq1 | 11 | 110 | 10 | 111 | 1 | 102 | 56.2 | 2.1E-15 |
| 38 | comp44017_c0_seq1 | 2 | 185 | 1 | 186 | 139 | 321 | 80.8 | 7E-23 |
| 39 | comp44650_c0_seq1 | 9 | 79 | 1 | 79 | 236 | 308 | 47 | 1.3E-12 |
| 40 | comp44661_c0_seq1 | 2 | 77 | 1 | 77 | 314 | 388 | 58.9 | 3E-16 |
| 41 | comp45726_c0_seq1 | 1 | 123 | 1 | 123 | 286 | 408 | 153.3 | 7.1E-45 |
| 42 | comp46180_c0_seq1 | 27 | 127 | 4 | 133 | 199 | 303 | 54.4 | 7.1E-15 |
| 43 | comp46521_c0_seq1 | 15 | 74 | 6 | 74 | 239 | 300 | 25.3 | 0.000005 |
| 44 | comp46894_c0_seq1 | 2 | 130 | 1 | 135 | 312 | 438 | 164.9 | 2.2E-48 |
| 45 | comp47341_c0_seq1 | 1 | 93 | 1 | 106 | 355 | 447 | 107.9 | 4.4E-31 |
| 46 | comp47563_c0_seq1 | 4 | 68 | 1 | 87 | 381 | 445 | 42.2 | 3.7E-11 |
| 47 | comp48174_c0_seq1 | 1 | 255 | 1 | 255 | 33 | 299 | 105.1 | 3E-30 |
| 48 | comp48310_c0_seq1 | 5 | 88 | 1 | 88 | 326 | 414 | 94.8 | 4E-27 |
| 49 | comp48462_c0_seq1 | 67 | 148 | 62 | 150 | 26 | 113 | 35.4 | 4.3E-09 |
| 50 | comp48997_c0_seq2 | 5 | 165 | 2 | 166 | 138 | 296 | 71.9 | 3.6E-20 |
| 51 | comp49282_c0_seq1 | 1 | 213 | 1 | 227 | 40 | 251 | 66.7 | 1.3E-18 |
| 52 | comp49510_c0_seq1 | 3 | 94 | 1 | 96 | 223 | 324 | 35.9 | 2.9E-09 |
| 53 | comp50117_c0_seq1 | 1 | 48 | 1 | 67 | 382 | 429 | 40.3 | 1.3E-10 |
| 54 | comp5023_c0_seq1 | 1 | 69 | 1 | 80 | 240 | 310 | 32.8 | 2.7E-08 |
| 55 | comp50251_c0_seq1 | 19 | 186 | 6 | 186 | 109 | 272 | 47.9 | 6.7E-13 |
| 56 | comp50663_c0_seq1 | 2 | 125 | 1 | 126 | 200 | 319 | 63.8 | 9.9E-18 |
| 57 | comp5110_c0_seq1 | 1 | 83 | 1 | 89 | 360 | 441 | 74.1 | 7.6E-21 |
| 58 | comp51512_c0_seq1 | 8 | 69 | 2 | 98 | 251 | 313 | 39.5 | 2.5E-10 |
| 59 | comp51913_c0_seq1 | 1 | 173 | 1 | 173 | 240 | 412 | 206 | 7.4E-61 |
| 60 | comp53557_c0_seq1 | 7 | 225 | 1 | 229 | 121 | 346 | 107.9 | 4.2E-31 |
| 61 | comp54366_c0_seq1 | 3 | 167 | 1 | 169 | 296 | 461 | 159.7 | 8.2E-47 |
| 62 | comp54370_c0_seq1 | 4 | 215 | 1 | 220 | 237 | 450 | 196 | 8.1E-58 |
| 63 | comp54412_c0_seq2 | 48 | 367 | 48 | 367 | 1 | 330 | 182.2 | 1.2E-53 |
| 64 | comp54621_c0_seq3 | 21 | 229 | 1 | 229 | 176 | 386 | 180.6 | 3.8E-53 |
| 65 | comp55183_c0_seq1 | 2 | 118 | 1 | 118 | 291 | 407 | 136.3 | 1.1E-39 |
| 66 | comp55183_c1_seq1 | 3 | 163 | 1 | 164 | 124 | 295 | 58.3 | 4.8E-16 |
| 67 | comp56111_c0_seq1 | 5 | 308 | 1 | 310 | 142 | 437 | 207.7 | 2.3E-61 |
| 68 | comp56178_c0_seq1 | 62 | 182 | 62 | 187 | 1 | 121 | 73.4 | 1.2E-20 |
| 69 | comp56356_c0_seq1 | 2 | 47 | 1 | 56 | 408 | 453 | 29.6 | 2.5E-07 |
| 70 | comp56533_c2_seq1 | 6 | 236 | 1 | 236 | 138 | 375 | 160.1 | 6.4E-47 |
| 71 | comp57416_c0_seq1 | 1 | 412 | 1 | 425 | 23 | 448 | 255.9 | 5.5E-76 |
| 72 | comp57607_c0_seq1 | 2 | 105 | 1 | 117 | 346 | 448 | 98.9 | 2.2E-28 |
| 73 | comp57607_c1_seq1 | 6 | 135 | 3 | 137 | 34 | 163 | 62 | 3.5E-17 |
| 74 | comp57607_c2_seq1 | 17 | 184 | 4 | 185 | 189 | 352 | 112.8 | 1.4E-32 |
| 75 | comp57864_c1_seq3 | 53 | 332 | 53 | 333 | 1 | 289 | 130.2 | 7.2E-38 |
| 76 | comp58033_c0_seq1 | 190 | 301 | 186 | 347 | 295 | 408 | 50 | 1.6E-13 |
| 77 | comp58195_c0_seq1 | 14 | 102 | 2 | 114 | 134 | 230 | 29.2 | 3.2E-07 |
| 78 | comp58714_c0_seq1 | 40 | 146 | 38 | 153 | 294 | 398 | 63.5 | 1.2E-17 |
| 79 | comp59102_c0_seq1 | 18 | 271 | 3 | 279 | 35 | 309 | 50.9 | 8.2E-14 |
| 80 | comp59726_c0_seq1 | 4 | 288 | 1 | 295 | 182 | 456 | 213.6 | 3.8E-63 |
| 81 | comp60247_c1_seq1 | 5 | 324 | 1 | 325 | 79 | 392 | 143.8 | 5.7E-42 |
| 82 | comp60480_c0_seq1 | 45 | 387 | 45 | 387 | 1 | 358 | 119 | 1.8E-34 |
| 83 | comp60568_c0_seq1 | 59 | 199 | 18 | 200 | 201 | 337 | 52.9 | 2.1E-14 |
| 84 | comp62039_c0_seq1 | 2 | 158 | 1 | 168 | 114 | 263 | 27.7 | 9.1E-07 |
| 85 | comp62160_c0_seq1 | 4 | 251 | 1 | 252 | 108 | 344 | 121.7 | 2.8E-35 |
| 86 | comp62406_c1_seq1 | 2 | 333 | 1 | 333 | 86 | 417 | 160.6 | 4.4E-47 |
| 87 | comp63045_c0_seq2 | 44 | 400 | 44 | 400 | 1 | 358 | 235 | 1.2E-69 |
| 88 | comp63226_c0_seq1 | 1 | 122 | 1 | 126 | 337 | 459 | 119.4 | 1.4E-34 |
| 89 | comp63234_c0_seq1 | 1 | 227 | 1 | 228 | 92 | 311 | 114 | 6.1E-33 |
| 90 | comp64738_c0_seq2 | 2 | 79 | 1 | 79 | 26 | 103 | 33.7 | 1.4E-08 |
| 91 | comp65334_c0_seq1 | 14 | 237 | 1 | 238 | 84 | 309 | 109.9 | 1E-31 |
| 92 | comp66429_c0_seq4 | 47 | 461 | 47 | 468 | 1 | 442 | 197.6 | 2.7E-58 |
| 93 | comp66651_c0_seq3 | 13 | 215 | 4 | 232 | 236 | 443 | 198.7 | 1.2E-58 |
| 94 | comp67739_c0_seq1 | 37 | 349 | 33 | 349 | 7 | 323 | 117 | 7.5E-34 |
| 95 | comp67799_c0_seq2 | 50 | 340 | 13 | 353 | 129 | 436 | 149.9 | 7.8E-44 |
| 96 | comp69093_c4_seq11 | 168 | 355 | 158 | 370 | 257 | 442 | 135.2 | 2.2E-39 |
| 97 | comp69457_c1_seq1 | 88 | 221 | 76 | 222 | 24 | 160 | 50.6 | 1E-13 |
| 98 | comp69457_c2_seq1 | 2 | 239 | 1 | 248 | 207 | 440 | 196.9 | 4.4E-58 |
| 99 | comp69493_c0_seq1 | 61 | 128 | 61 | 136 | 1 | 70 | 35.2 | 4.7E-09 |
| 100 | comp69666_c1_seq1 | 10 | 53 | 5 | 68 | 397 | 440 | 58.3 | 4.9E-16 |
| 101 | comp70731_c0_seq1 | 90 | 169 | 64 | 169 | 21 | 105 | 39.3 | 2.8E-10 |
| 102 | comp70970_c0_seq1 | 1 | 197 | 1 | 206 | 29 | 230 | 69.2 | 2.4E-19 |
| 103 | comp71366_c0_seq1 | 1 | 102 | 1 | 103 | 282 | 377 | 71.4 | 5E-20 |
| 104 | comp71698_c0_seq1 | 20 | 129 | 8 | 129 | 175 | 285 | 44.8 | 5.7E-12 |
| 105 | comp72521_c0_seq1 | 1 | 87 | 1 | 91 | 355 | 441 | 92.8 | 1.6E-26 |
| 106 | comp72640_c0_seq1 | 8 | 123 | 2 | 125 | 198 | 310 | 79 | 2.5E-22 |
| 107 | comp72680_c0_seq1 | 2 | 74 | 1 | 74 | 300 | 373 | 75.1 | 3.7E-21 |
| 108 | comp72930_c0_seq1 | 2 | 83 | 1 | 85 | 46 | 127 | 25.5 | 4.1E-06 |
| 109 | comp73146_c0_seq1 | 6 | 65 | 2 | 67 | 263 | 323 | 46.7 | 1.6E-12 |
| 110 | comp73330_c0_seq1 | 5 | 77 | 4 | 81 | 379 | 456 | 54.1 | 9.1E-15 |
| 111 | comp73864_c0_seq1 | 1 | 102 | 1 | 112 | 354 | 453 | 101 | 5.4E-29 |
| 112 | comp73985_c0_seq1 | 4 | 83 | 1 | 84 | 299 | 379 | 91.8 | 3.3E-26 |
| 113 | comp7584_c0_seq1 | 7 | 96 | 2 | 97 | 214 | 308 | 40.2 | 1.4E-10 |
| 114 | comp7665_c0_seq1 | 13 | 83 | 5 | 83 | 319 | 388 | 43.2 | 1.8E-11 |
| 115 | comp78074_c0_seq1 | 5 | 82 | 1 | 82 | 310 | 387 | 82.2 | 2.6E-23 |
| 116 | comp78605_c0_seq1 | 17 | 101 | 2 | 106 | 81 | 162 | 35.5 | 3.9E-09 |
| 117 | comp79716_c0_seq1 | 3 | 82 | 1 | 99 | 354 | 437 | 88.8 | 2.6E-25 |
| 118 | comp80698_c0_seq1 | 5 | 158 | 2 | 160 | 120 | 278 | 49.2 | 2.7E-13 |
| 119 | comp80750_c0_seq1 | 1 | 66 | 1 | 81 | 382 | 446 | 49.1 | 2.9E-13 |
| 120 | comp81246_c0_seq1 | 1 | 81 | 1 | 93 | 354 | 439 | 76.4 | 1.5E-21 |
| 121 | comp81912_c0_seq1 | 8 | 54 | 2 | 70 | 400 | 446 | 44.6 | 6.9E-12 |
| 122 | comp83040_c0_seq1 | 1 | 81 | 1 | 104 | 354 | 437 | 80.9 | 6.6E-23 |
| 123 | comp85463_c0_seq1 | 2 | 69 | 1 | 69 | 31 | 99 | 32.8 | 2.5E-08 |
| 124 | comp86860_c0_seq1 | 6 | 68 | 2 | 69 | 238 | 300 | 49.5 | 2.2E-13 |
| 125 | comp90024_c0_seq1 | 2 | 109 | 1 | 109 | 221 | 325 | 83 | 1.5E-23 |
| 126 | comp91713_c0_seq1 | 6 | 138 | 1 | 139 | 178 | 305 | 69 | 2.8E-19 |
| 127 | comp91971_c0_seq1 | 5 | 60 | 1 | 68 | 251 | 307 | 31.4 | 6.6E-08 |
| 128 | comp92134_c0_seq1 | 6 | 93 | 2 | 98 | 197 | 292 | 36 | 2.7E-09 |
| 129 | comp92856_c0_seq1 | 1 | 64 | 1 | 68 | 326 | 389 | 58.5 | 4E-16 |
| 130 | comp94231_c0_seq1 | 2 | 74 | 1 | 75 | 264 | 333 | 73.1 | 1.6E-20 |
| 131 | comp94322_c0_seq1 | 15 | 68 | 1 | 72 | 257 | 310 | 54.6 | 6.4E-15 |
| 132 | comp94501_c0_seq1 | 2 | 106 | 1 | 111 | 344 | 445 | 108.3 | 3.2E-31 |
| 133 | comp96408_c0_seq1 | 3 | 69 | 1 | 72 | 242 | 309 | 42.2 | 3.7E-11 |
| 134 | comp98652_c0_seq1 | 2 | 67 | 1 | 67 | 323 | 389 | 67.3 | 9E-19 |
| 135 | comp99171_c0_seq1 | 1 | 53 | 1 | 58 | 408 | 458 | 30.6 | 1.2E-07 |
