# Supplementary material for: Computational Identification and Systematic Classification of Novel Cytochrome P450 Genes in Salvia miltiorrhiza
Source: PLoS One. 2014 Dec 10;9(12):e115149. doi: 10.1371/journal.pone.0115149 (PMC4262458; doi:10.1371/journal.pone.0115149)
Supplement: S4 Table — Expression levels (FPKM) of full-length CYP450 genes across three tissues in S. miltiorrhiza and the comparison of their expression profiles to that of the marker gene CYP76AH1. (DOC) [file pone.0115149.s004.doc]

**Table S4 Expression levels (FPKM) of full-length CYP450 genes across three tissues in *S. miltiorrhiza* and comparison of their expression profiles to that of the marker gene, CYP76AH1*.***

| CYP450 Gene Name | Expression levels (FPKM) | | | Pearson correlation with SmCYP76AH1 |
| --- | --- | --- | --- | --- |
|  | Flower | Leaf | Root |  |
| SmCYP76AH1 | 0.97 | 3.08 | 39.01 | 1 |
| SmCYP92A73 | 22.14 | 24.23 | 64.23 | 0.999986 |
| SmCYP704A99 | 2.43 | 3.95 | 35.3 | 0.999965 |
| SmCYP98A75 | 357.32 | 388.85 | 1127.86 | 0.999913 |
| SmCYP98A77 | 24.58 | 29.06 | 163.43 | 0.99978 |
| SmCYP716C12 | 12.97 | 19.08 | 481.85 | 0.999278 |
| SmCYP81B61 | 1.12 | 1.42 | 34.58 | 0.999136 |
| SmCYP74A1 | 3.5 | 4.02 | 67.11 | 0.999107 |
| SmCYP71D411 | 0 | 0.1 | 60.1 | 0.998852 |
| SmCYP98A78 | 441.56 | 569.82 | 1579.63 | 0.998559 |
| SmCYP72A329 | 2.31 | 11.54 | 83.92 | 0.998538 |
| SmCYP79D40 | 2.34 | 2.14 | 22.52 | 0.998324 |
| SmCYP94C54 | 8.15 | 2.88 | 358.8 | 0.998061 |
| SmCYP716D25 | 8.26 | 10.33 | 24.09 | 0.997465 |
| SmCYP749A39 | 15.01 | 26.17 | 89.08 | 0.995879 |
| SmCYP707A102 | 52.04 | 38.07 | 328.91 | 0.995774 |
| SmCYP81Q40 | 0.72 | 0.03 | 11.39 | 0.994642 |
| SmCYP714A25 | 4.28 | 7.61 | 23.97 | 0.994035 |
| SmCYP71AT93 | 2.21 | 0.21 | 29.94 | 0.994002 |
| SmCYP711A44 | 2.44 | 6.79 | 26.14 | 0.992335 |
| SmCYP736A122 | 6.17 | 11.67 | 33.42 | 0.989842 |
| SmCYP728D17 | 3.17 | 6.47 | 19.39 | 0.989604 |
| SmCYP78A114 | 1.59 | 3.8 | 11.56 | 0.986702 |
| SmCYP73A120 | 437.56 | 661.57 | 1445.77 | 0.986613 |
| SmCYP98A76 | 115.15 | 92 | 270.96 | 0.985825 |
| SmCYP749A37 | 2.06 | 0.42 | 12.11 | 0.98397 |
| SmCYP76A36 | 6.2 | 1.67 | 29.59 | 0.979846 |
| SmCYP94B50 | 22.88 | 19.33 | 38.64 | 0.975254 |
| SmCYP76AK2 | 3.03 | 4.8 | 9.03 | 0.970909 |
| SmCYP94C55 | 3.18 | 1.73 | 8.66 | 0.969148 |
| SmCYP71AU53 | 2.05 | 1.27 | 4.06 | 0.948056 |
| SmCYP94A48 | 13.6 | 3.89 | 37.73 | 0.945479 |
| SmCYP82V2 | 3.79 | 8.92 | 16.8 | 0.938411 |
| SmCYP84A60 | 2.74 | 7.85 | 15.46 | 0.935458 |
| SmCYP720A1 | 0.67 | 0.36 | 1.33 | 0.933199 |
| SmCYP71D374 | 1.56 | 24.87 | 55.43 | 0.922346 |
| SmCYP76G16 | 69.74 | 109.61 | 143.84 | 0.868738 |
| SmCYP81C16 | 7.36 | 0.64 | 14.36 | 0.846626 |
| SmCYP72A326 | 23.2 | 2.84 | 41.22 | 0.820706 |
| SmCYP72A328 | 5.43 | 15.85 | 19.85 | 0.747949 |
| SmCYP716A89 | 35.89 | 193.67 | 225.34 | 0.666606 |
| SmCYP84A61 | 6.55 | 2.79 | 7.81 | 0.657773 |
| SmCYP76AK3 | 4.37 | 10.69 | 10.88 | 0.563495 |
| SmCYP701A40 | 45.82 | 12.97 | 48.27 | 0.511061 |
| SmCYP85A1 | 8.05 | 4.65 | 8.27 | 0.504204 |
| SmCYP82D70 | 0.86 | 26.42 | 22.98 | 0.433746 |
| SmCYP714G14 | 51.24 | 96.07 | 84.83 | 0.323692 |
| SmCYP94D47 | 12.05 | 52.18 | 38.9 | 0.239813 |
| SmCYP71BE37 | 18.88 | 7.45 | 16.13 | 0.239318 |
| SmCYP81Q43 | 2.15 | 33.73 | 21.65 | 0.183157 |
| SmCYP749A40 | 3.65 | 1.94 | 3.11 | 0.159533 |
| SmCYP78A115 | 0.1 | 2.14 | 1.02 | -0.00718 |
| SmCYP72A327 | 61.96 | 118.69 | 86.19 | -0.03459 |
| SmCYP76S7 | 2.04 | 22.56 | 9.39 | -0.1127 |
| SmCYP714G13 | 54.18 | 360.2 | 147.67 | -0.17071 |
| SmCYP749A38 | 0.89 | 3.66 | 1.7 | -0.18482 |
| SmCYP92B28 | 0.25 | 14.05 | 4.27 | -0.18602 |
| SmCYP721A38 | 7.61 | 20.33 | 11.16 | -0.19906 |
| SmCYP71AP14 | 2.28 | 29.8 | 8.09 | -0.26923 |
| SmCYP96A84 | 0.34 | 1.18 | 0.49 | -0.30127 |
| SmCYP81Q41 | 5.81 | 28.13 | 8.21 | -0.36733 |
| SmCYP75B80 | 27.03 | 3.73 | 8.21 | -0.38091 |
| SmCYP71AT92 | 18.08 | 120.02 | 26.49 | -0.38928 |
| SmCYP71AT91 | 3.01 | 38.93 | 5.18 | -0.40807 |
| SmCYP71AT90 | 0.02 | 4.78 | 0.18 | -0.43013 |
| SmCYP707A100 | 0.65 | 33.88 | 0.18 | -0.46744 |
| SmCYP71AU52 | 1.86 | 17.6 | 1.54 | -0.47209 |
| SmCYP97B34 | 4.77 | 26.89 | 4.19 | -0.47648 |
| SmCYP71A57 | 5.5 | 101.08 | 1.43 | -0.48846 |
| SmCYP71A59 | 3.88 | 43.06 | 1.15 | -0.50767 |
| SmCYP75B79 | 10.54 | 119.95 | 1.39 | -0.51725 |
| SmCYP706C35 | 47.22 | 14.49 | 15.47 | -0.51983 |
| SmCYP72A331 | 11.49 | 56.43 | 7 | -0.52818 |
| SmCYP706G11 | 13.75 | 99.4 | 4.32 | -0.53488 |
| SmCYP97C28 | 14.4 | 95.56 | 4.59 | -0.54184 |
| SmCYP75A57 | 106.01 | 0.05 | 0 | -0.54247 |
| SmCYP82U4 | 9.3 | 76.58 | 0.08 | -0.55207 |
| SmCYP72A330 | 43.41 | 185.83 | 23.25 | -0.55488 |
| SmCYP71D410 | 15.27 | 121.12 | 0.1 | -0.55597 |
| SmCYP704B37 | 14.22 | 0.48 | 0 | -0.56686 |
| SmCYP81B62 | 6.12 | 36.94 | 0 | -0.58867 |
| SmCYP71AU51 | 24.56 | 8.01 | 6.56 | -0.60162 |
| SmCYP81Q42 | 2.2 | 11.52 | 0.11 | -0.60289 |
| SmCYP71D412 | 13.5 | 59.93 | 2.98 | -0.60415 |
| SmCYP96A85 | 104 | 29.18 | 22.2 | -0.60519 |
| SmCYP707A99 | 2.17 | 11 | 0 | -0.61434 |
| SmCYP93B25 | 57.58 | 10.51 | 2.36 | -0.65191 |
| SmCYP76AK3 | 0.69 | 2.32 | 0.15 | -0.65605 |
| SmCYP71A58 | 5.15 | 16.29 | 1.13 | -0.66913 |
| SmCYP97A41 | 31.03 | 73.95 | 15.08 | -0.67374 |
| SmCYP92B29 | 31.61 | 95.04 | 5.89 | -0.68769 |
| SmCYP736A123 | 5.31 | 14.37 | 0.17 | -0.7445 |
| SmCYP90C19 | 8.53 | 14.9 | 4.36 | -0.7694 |
| SmCYP734A33 | 3.32 | 5.31 | 1.7 | -0.80681 |
| SmCYP71AH15 | 2.01 | 4.24 | 0 | -0.82369 |
| SmCYP77A27 | 12.02 | 23.3 | 0.42 | -0.84465 |
| SmCYP89A115 | 20.95 | 29.1 | 11.73 | -0.85903 |
| SmCYP76A35 | 30.12 | 55.22 | 1.58 | -0.85978 |
| SmCYP704A98 | 10.86 | 19.76 | 0 | -0.86992 |
| SmCYP82D71 | 6.28 | 10.42 | 0.46 | -0.88889 |
| SmCYP51G1 | 88.62 | 110.34 | 55.89 | -0.89749 |
| SmCYP74B21 | 28.2 | 44.37 | 1.01 | -0.9101 |
| SmCYP86A92 | 66.57 | 104.58 | 0.94 | -0.91297 |
| SmCYP78A113 | 9.28 | 5.61 | 1.16 | -0.91357 |
| SmCYP736A121 | 19.67 | 29.9 | 0.3 | -0.9224 |
| SmCYP714E21 | 2.89 | 2.14 | 1.08 | -0.93026 |
| SmCYP77A28 | 71.82 | 105.69 | 0 | -0.93285 |
| SmCYP90B26 | 10.15 | 11.37 | 3.58 | -0.98096 |
| SmCYP71D413 | 6.6 | 7.13 | 2.72 | -0.98728 |
| SmCYP94A49 | 23.34 | 18.6 | 0.04 | -0.98961 |
| SmCYP727B10 | 26.25 | 22 | 2.89 | -0.99254 |
| SmCYP88A52 | 6.08 | 5.25 | 0.88 | -0.99503 |
| SmCYP71AT89 | 6.81 | 5.79 | 0 | -0.99596 |
| SmCYP90A39 | 22.07 | 22.5 | 1.25 | -0.99775 |
| SmCYP86A91 | 28.3 | 25.44 | 0.47 | -0.99902 |
| SmCYP707A101 | 5.11 | 4.71 | 1.02 | -0.99922 |
